# Trends in Maternal Mortality and Severe Maternal Morbidity During Delivery-Related Hospitalizations in the United States, 2008 to 2021

**DOI:** 10.1001/jamanetworkopen.2023.17641

**Published:** 2023-06-22

**Authors:** Dorothy A. Fink, Deborah Kilday, Zhun Cao, Kelly Larson, Adrienne Smith, Craig Lipkin, Raymond Perigard, Richelle Marshall, Taryn Deirmenjian, Ashley Finke, Drew Tatum, Ning Rosenthal

**Affiliations:** 1 Office on Women’s Health, US Department of Health & Human Services, Washington, DC; 2 Premier Inc, Charlotte, North Carolina; 3 Now with Office on Women’s Health, US Department of Health & Human Services, Washington, DC; 4 Now with Attentive Mobile, New York, New York

## Abstract

**Importance:**

Maternal mortality and severe maternal morbidity (SMM) are important focus areas in public health. Further understanding trends, health disparities, and risk factors for these adverse outcomes is vital to public health decision-making.

**Objective:**

To describe trends and risk factors for delivery-related maternal deaths and SMM in the United States.

**Design, Setting, and Participants:**

This is a retrospective cross-sectional study using data from a large, geographically diverse, all-payer hospital administrative database. Hospital discharges from January 2008 to December 2021 with any Medicare Severity Diagnosis Related Group, *International Classification of Diseases, Ninth Revision, Clinical Modification*, or *International Classification of Diseases, Tenth Revision, Clinical Modification *delivery diagnosis or procedure code were included. Data analysis took place from February 2021 to March 2023.

**Exposures:**

Year, quarter (Q), age, race and ethnicity, delivery method.

**Main Outcomes and Measures:**

Maternal mortality, SMM during delivery-related hospitalization.

**Results:**

Overall, 11 628 438 unique hospital discharges were analyzed, with a mean (SD) age of 28 (6) years. There were 437 579 (3.8%) Asian, 92 547 (0.8%) American Indian, 1 640 355 (14.1%) Black, 1 762 392 (15.2%) Hispanic, 83 189 (0.7%) Pacific Islander, and 6 194 139 (53.3%) White patients. Regression-adjusted maternal mortality per 100 000 discharges declined from 10.6 deaths in Q1 2008 to 4.6 deaths in Q4 2021. Mortality was significantly higher among patients with advanced maternal age (eg, age 35-44 years vs 25-34 years: adjusted odds ratio [aOR], 1.49; 95% CI, 1.22-1.84). Other significant risk factors for mortality included cesarean delivery, comorbid conditions, complications, and COVID-19 diagnosis (eg, cesarean delivery: aOR, 2.28; 95% CI, 1.87-2.79). The prevalence of any SMM increased from 146.8 per 10 000 discharges in Q1 of 2008 to 179.8 per 10 000 discharges in Q4 of 2021. SMM risk factors included age 24 years or younger or age 35 years or older, belonging to a racial or ethnic minority group, cesarean delivery, Medicaid insurance, and having 1 or more comorbidities (eg, age 10-19 years: aOR, 1.39; 95% CI, 1.36-1.42).

**Conclusions and Relevance:**

This cross-sectional study found that delivery-related mortality in US hospitals decreased for all racial and ethnic groups, age groups, and modes of delivery during 2008 to 2021, likely demonstrating the impact of national strategies focused on improving maternal quality of care provided during delivery-related hospitalizations. SMM prevalence increased for all patients, with higher rates for racial and ethnic minority patients of any age. Advanced maternal age, racial or ethnic minority group status, cesarean delivery, and comorbidities were associated with higher odds of mortality and SMM.

## Key Points

**Question:**

What were trends of and risk factors associated with maternal mortality and severe maternal morbidity (SMM) among women giving birth in US hospitals during 2008 to 2021?

**Findings:**

In this cross-sectional study of more than 11.6 million delivery-related hospitalizations, regression-adjusted in-hospital maternal delivery-related mortality per 100 000 discharges declined from 10.6 to 4.6, while the prevalence of SMM per 10 000 discharges increased from 146.8 to 179.8 during 2008 to 2021. Differences were found across racial and ethnic groups, age, mode of delivery, and comorbidities for mortality and SMM.

**Meaning:**

In this study, in-hospital maternal mortality improved between 2008 and 2021 despite increases in SMM prevalence and presence of comorbidities for the overall population.

## Teaser

This cross-sectional study describes trends of and risk factors associated with delivery-related maternal deaths and severe maternal morbidity.

## Introduction

Complications from pregnancy and childbirth are leading contributors to mortality and severe morbidities, resulting in significant burden on pregnant patients and their babies. Among developed countries, the United States has the highest maternal mortality ratio.^1^ In 2019, there were 3 747 540 births in the United States, with an estimated birth rate of 11.4 per 1000 population.^2^ According to US Pregnancy Mortality Surveillance System (PMSS) data, the pregnancy-related mortality ratio in the United States had increased since 1987 from 7.2 deaths per 100 000 live births to 17.3 deaths per 100 000 live births in 2017, although the trend slowed substantially after 2008.^3^

Maternal mortality has been described as the “tip of the iceberg” and maternal morbidity as a larger problem, “the base.”^4^ For every individual who dies as a result of their pregnancy, it is estimated that 20 or 30 more experience significant lifelong complications that affect their health and well-being.^5,6^ Severe maternal morbidity (SMM), which the US Centers for Disease Control and Prevention (CDC) defines as “unexpected outcomes of labor and delivery that result in significant short- or long-term consequences to a woman’s health,”^1^ has steadily increased in the United States in recent years and is estimated to affect more than 50 000 patients annually.

Causes of maternal deaths and SMM at the time of delivery are multifactorial and are not well documented.^7^ Measuring specific outcomes occurring during delivery and hospitalization could improve understanding of how to predict, manage, and mitigate maternal outcomes. In addition, enhanced understanding of the causes of delivery-related death and SMM can inform potential strategies to improve overall maternal health outcomes in the United States. This study aimed to provide evidence to enhance understanding of patterns, trends, and risk factors associated with delivery-related deaths and SMM in US hospitals using a large maternal sample in the hospital setting.

## Methods

### Study Design

This retrospective cross-sectional study was conducted to examine trends associated with delivery-related maternal in-hospital mortality and SMM between January 2008 and December 2021, using data from the Premier PINC AI Healthcare Database (PHD). All data were statistically deidentified and adherent to the Health Insurance Portability and Accountability Act. Based on US Title 45 Code of Federal Regulations, Part 46, this study was exempted from institutional review board approval and informed consent. The study followed the Strengthening the Reporting of Observational Studies in Epidemiology (STROBE) reporting guideline.

### Data Source

The PHD is a large, all-payer (including Medicaid), geographically diverse administrative database comprising more than 1200 US hospitals and health systems.^8^ This database represents approximately 25% of all US inpatient admissions. All data were validated at both facility and patient levels. The CDC, the National Institute of Health, and academic and industry researchers have used PHD data for studies in a variety of disease areas.^9,10,11,12,13,14,15^

### Study Population

This study reviewed inpatient hospitalizations between January 1, 2008, and December 31, 2021, with any Medicare Severity Diagnosis Related Group (MS-DRG) or *International Classification of Diseases, Ninth Revision, Clinical Modification* (*ICD-9-CM*) diagnosis or procedure codes (on or before September 30, 2015) or *International Classification of Diseases and Related Health Problems, Tenth Revision, Clinical Modification* (*ICD-10-CM*) codes (on or after October 1, 2015) indicating delivery (eTable 1 in Supplement 1). Hospitalizations for patients younger than 10 years at time of admission and those with evidence of abortive outcomes were excluded from the study. The index date was defined as the discharge date for the qualifying hospitalization. Missing data for categorical variables were included in the other or unknown group. Only a small percentage of patients had missing data, which should not have affected the trend analysis.

### Study Variables

#### SMM

Complications or procedures indicative of SMMs were examined during the delivery-related hospitalization; these included acute myocardial infarction, acute kidney failure, amniotic fluid embolism, aneurysm, cardiac arrest or ventricular fibrillation, cardioversion, disseminated intravascular coagulation, eclampsia, heart failure or arrest during procedure, puerperal cerebrovascular disorders, acute heart failure or pulmonary edema, severe anesthesia complications, sepsis, shock, sickle cell anemia with crisis, air and thrombotic embolism, blood transfusion, hysterectomy, temporary tracheostomy, and ventilation. The diagnosis and procedure codes to identify the complications are listed in eTable 2 in Supplement 1. The presence of any SMM was used as a measure for the adverse event occurring during delivery. Morbidities were reported as number of patients with each SMM or any SMM of interest per 10 000 eligible discharges.

#### In-Hospital Delivery-Related Mortality

Death was defined as having delivery-related hospitalization discharge status as deceased. In-hospital mortality was reported as the number of patients who died during index hospitalization per 100 000 eligible discharges.

### Patient, Hospital, and Visit Characteristics

Patient characteristics included age (10-19, 20-24, 25-34, 35-44, ≥45 years), race and ethnicity (categorized as American Indian, Asian, Black, Hispanic, Pacific Islander, White, and other or unknown), and primary insurance payer. The other or unknown category captures all patients who selected other category for race, had missing data for race or ethnicity, or had a hospital-reported race that could not be matched to the standard race categories used in this article. Race and ethnicity were reported by the hospital. For the purposes of this study, we defined racial or ethnic minority patients as those with race or ethnicity classifications other than White. Hospital characteristics included population served (urban, rural), teaching status, US census divisions (ie, Middle Atlantic, Mountain, East North Central, East South Central, New England, Pacific, South Atlantic, West North Central, and West South Central), and hospital size (1-299, 300-499, and ≥500 beds). Visit information, such as index year, quarter (Q), admission type (elective, emergency, urgent, or trauma center), and an indicator for pre–*ICD-10-CM* or post–*ICD-10-CM* coding system change on October 1, 2015, were also examined.

### Clinical Characteristics

The individual conditions in the Maternal Comorbidity Index (MCI)^16^ were assessed as potential risk factors of maternal mortality or morbidity, including pulmonary hypertension, placenta previa, sickle cell disease, gestational hypertension, mild or unspecified preeclampsia, severe preeclampsia, chronic kidney disease, preexisting hypertension, chronic ischemic heart disease, congenital heart disease, systemic lupus erythematosus, HIV, multiple gestation, substance use disorder, alcohol abuse, tobacco use, cardiac valvular disease, chronic congestive heart failure, asthma, preexisting diabetes, gestational diabetes, obesity, cystic fibrosis, and previous cesarean delivery (eTable 3 in Supplement 1). The type of delivery (vaginal, cesarean) and COVID-19 status were also examined.

All SMMs were included as covariates for the mortality analysis. Because of overlap across comorbid conditions, certain SMMs were grouped together. Per CDC recommendations, cardiac conditions (including acute myocardial infarction, cardiac arrest or ventricular fibrillation, conversion of cardiac rhythm, heart failure or arrest during surgery or procedure, and pulmonary edema or acute heart failure) were grouped into 1 binary variable called cardiovascular complications for multivariable modeling. Acute respiratory conditions (including acute respiratory distress syndrome, temporary tracheostomy, and ventilation) were grouped into a binary variable called respiratory complications.^17^ Hemorrhage and blood transfusion were combined into bleeding complications with 3 categories: no bleeding, hemorrhage with no blood transfusion, and blood transfusion. Eclampsia, severe preeclampsia without eclampsia, mild or unspecified preeclampsia without eclampsia, or severe preeclampsia, and no preeclampsia or eclampsia were grouped into one 4-level covariate.

### Statistical Analysis

Descriptive analysis was performed to assess the distribution of demographics and hospital and clinical characteristics for each year. Categorical variables were expressed as counts and percentages. Owing to space limitations, we only included specific descriptive results for 2008, 2014 (ie, the year before the *ICD-9-CM* to *ICD-10-CM* coding change), 2016 (ie, the year after the *ICD-9-CM* to *ICD-10-CM* coding change), 2019 (ie, the year before the COVID-19 pandemic), 2020, and 2021 (ie, years during the COVID-19 pandemic), rather than for all years in this study.

Two separate multivariable logistic regression models were created to assess the independent associations of potential risk factors with delivery-related maternal mortality and SMM, adjusting for confounders. For both models, patient demographics, hospital and visit characteristics, and MCI conditions were included as covariates. In the mortality regression, the SMM complications were added to the model to account for disease conditions that happened during the delivery-related hospitalization before the occurrence of mortality. In addition, a logistic regression of mortality without SMMs as covariates was performed as a sensitivity analysis. Backward selection with *P* < .05 was used to select final models, with the exception that patient age, race and ethnicity, delivery type, and study year and Q were kept in the model regardless of *P* values. For the mortality model, SMM conditions that were closely related to each other were combined. Combined variables included bleeding complications, cardiovascular complications, respiratory complications, and an eclampsia or preeclampsia category. In the regression of SMM, eclampsia was 1 component of the SMM outcome, while the preeclampsia conditions were used as separate covariates in the model.

Adjusted mortality and SMM rates for the overall study population were calculated using recycled prediction methods^16,18^ based on estimates from the regressions. Adjusted mortality and SMM rates were also reported by age group, race and ethnicity, and type of delivery, based on additional regression models that included interaction terms between year and the variable of interest.

All analyses were conducted using Python Scikit-Learn package version 0.22.1 (Python Software Foundation). Analysis of the data took place from February 2021 through March 2023. *P* values were 2-sided, and statistical significance was set at *P* < .05.

## Results

### Patient Characteristics

Among the 11 628 438 eligible discharges related to delivery, more than half (6 498 217 [55.9%]) were among patients aged 25 to 34 years, 1 885 571 (16.2%) were among patients aged 35 years or older, and 759 301 (6.5%) were among patients aged 10 to 19 years. There were 437 579 (3.8%) Asian patients, 92 547 (0.8%) American Indian patients, 1 640 355 (14.1%) Black patients, 1 762 392 (15.2%) Hispanic patients, 83 189 (0.7%) Pacific Islander patients, and 6 194 139 (53.3%) White patients. Medicaid was identified as the primary payer for 4 958 174 discharges (42.6%). The census region distribution reflected the geographic distribution of the PHD patient population. Approximately one-third of the sample underwent cesarean delivery. The proportion of discharges in younger age groups decreased while the proportion in older age groups increased over the study period. The distribution of race and ethnicity, primary payer type, census region, and delivery type did not differ significantly across years (Table 1).

**Table 1.** Demographic and Clinical Characteristics of Hospital Inpatient Discharges for Newborn Delivery From 2008 to 2021

Characteristic	Overall (2008-2021) (N = 11 628 438)	2008 (n = 545 297)	2014 (n = 959 874)	2016 (n = 947 865)	2019 (n = 895 086)	2020 (n = 816 259)	2021 (n = 737 241)
**Demographic characteristics, No. (%)**							
Age group, y							
10-19	759 301 (6.5)	54 859 (10.1)	61 862 (6.4)	50 844 (5.4)	43 283 (4.8)	37 984 (4.7)	31 551 (4.3)
20-24	2 485 349 (21.4)	131 265 (24.1)	214 382 (22.3)	193 547 (20.4)	172 562 (19.3)	155 545 (19.1)	135 711 (18.4)
25-34	6 498 217 (55.9)	280 481 (51.4)	537 605 (56.0)	546 059 (57.6)	517 947 (57.9)	472 928 (57.9)	430 853 (58.4)
35-44	1 863 674 (16.0)	77 861 (14.3)	144 381 (15.0)	155 624 (16.4)	159 443 (17.8)	147 978 (18.1)	137 560 (18.7)
≥45	21 897 (0.2)	831 (0.2)	1644 (0.2)	1791 (0.2)	1851 (0.2)	1824 (0.2)	1566 (0.2)
Race and ethnicity							
American Indian	92 547 (0.8)	3614 (0.7)	7400 (0.8)	7031 (0.7)	7078 (0.8)	6452 (0.8)	5998 (0.8)
Asian	437 579 (3.8)	97 (<0.1)	40 970 (4.3)	45 423 (4.8)	38 467 (4.3)	37 268 (4.6)	33 805 (4.6)
Black	1 640 355 (14.1)	76 464 (14.0)	127 521 (13.3)	130 219 (13.7)	130 805 (14.6)	121 362 (14.9)	107 857 (14.6)
Hispanic	1 762 392 (15.2)	71 490 (13.1)	133 203 (13.9)	127 114 (13.4)	149 926 (16.7)	146 098 (17.9)	142 558 (19.3)
Pacific Islander	83 189 (0.7)	2 (<0.1)	7432 (0.8)	7584 (0.8)	7236 (0.8)	6745 (0.8)	5991 (0.8)
White	6 194 139 (53.3)	267 502 (49.1)	516 803 (53.8)	539 920 (57.0)	483 398 (54.0)	437 934 (53.7)	396 643 (53.8)
Other or unknown	1 418 237 (12.2)	126 128 (23.1)	126 545 (13.2)	90 574 (9.6)	78 176 (8.7)	60 400 (7.4)	44 389 (6.0)
Payer							
Medicaid	4 958 174 (42.6)	225 608 (41.4)	408 037 (42.5)	395 360 (41.7)	382 978 (42.8)	352 971 (43.2)	314 442 (42.7)
Commercial	5 828 008 (50.1)	276 056 (50.6)	481 244 (50.1)	485 290 (51.2)	451 857 (50.5)	409 232 (50.1)	376 045 (51.0)
Charity or indigent	18 816 (0.2)	1076 (0.2)	1792 (0.2)	1711 (0.2)	1240 (0.1)	964 (0.1)	706 (0.1)
Other	823 440 (7.1)	42 557 (7.8)	68 801 (7.2)	65 504 (6.9)	59 011 (6.6)	53 092 (6.5)	46 048 (6.2)
Census region							
West North Central	706 715 (6.1)	31 165 (5.7)	62 499 (6.5)	64 931 (6.9)	58 928 (6.6)	48 407 (5.9)	45 065 (6.1)
South Atlantic	3 134 865 (27.0)	154 102 (28.3)	241 336 (25.1)	244 261 (25.8)	234 747 (26.2)	217 066 (26.6)	184 555 (25.0)
East South Central	782 615 (6.7)	24 419 (4.5)	74 856 (7.8)	69 372 (7.3)	65 104 (7.3)	62 533 (7.7)	60 427 (8.2)
East North Central	1 639 978 (14.1)	62 432 (11.4)	135 177 (14.1)	134 357 (14.2)	145 291 (16.2)	135 827 (16.6)	127 165 (17.2)
Middle Atlantic	1 385 382 (11.9)	75 875 (13.9)	94 350 (9.8)	107 311 (11.3)	118 432 (13.2)	101 991 (12.5)	85 193 (11.6)
Pacific	1 585 909 (13.6)	100 431 (18.4)	136 760 (14.2)	153 325 (16.2)	79 905 (8.9)	74 353 (9.1)	68 644 (9.3)
West South Central	1 411 885 (12.1)	59 814 (11.0)	122 417 (12.8)	108 469 (11.4)	112 469 (12.6)	97 420 (11.9)	85 263 (11.6)
Mountain	705 575 (6.1)	25 806 (4.7)	68 574 (7.1)	44 687 (4.7)	63 908 (7.1)	62 070 (7.6)	63 692 (8.6)
New England	275 514 (2.4)	11 253 (2.1)	23 905 (2.5)	21 152 (2.2)	16 302 (1.8)	16 592 (2.0)	17 237 (2.3)
Delivery type							
Vaginal	7 748 427 (66.6)	359 909 (66.0)	641 973 (66.9)	629 087 (66.4)	604 494 (67.5)	547 666 (67.1)	493 125 (66.9)
Cesarean	3 830 448 (32.9)	184 583 (33.8)	316 392 (33.0)	306 935 (32.4)	285 510 (31.9)	263 072 (32.2)	239 587 (32.5)
Unknown	49 563 (0.4)	805 (0.1)	1509 (0.2)	11 843 (1.2)	5082 (0.6)	5521 (0.7)	4529 (0.6)
**Maternal comorbid conditions, No. (rate per 1000 discharges)**							
Pulmonary hypertension	2692 (0.2)	115 (0.2)	205 (0.2)	177 (0.2)	232 (0.3)	220 (0.3)	233 (0.3)
Placenta previa	70 431 (6.1)	2976 (5.5)	5537 (5.8)	5894 (6.2)	5934 (6.6)	5639 (6.9)	5177 (7.0)
Sickle cell disease	48 352 (4.2)	720 (1.3)	1922 (2.0)	5461 (5.8)	6375 (7.1)	6256 (7.7)	5536 (7.5)
Gestational hypertension	584 415 (50.3)	17 728 (32.5)	39 796 (41.5)	47 693 (50.3)	60 811 (67.9)	60 194 (73.7)	60 685 (82.3)
Mild preeclampsia or unspecified preeclampsia	329 904 (28.4)	14 760 (27.1)	28 488 (29.7)	22 786 (24.0)	26 110 (29.2)	24 839 (30.4)	23 182 (31.4)
Severe preeclampsia	342 672 (29.5)	9888 (18.1)	23 600 (24.6)	27 187 (28.7)	36 222 (40.5)	35 129 (43)	35 278 (47.9)
Chronic kidney disease	23 557 (2.0)	1293 (2.4)	2815 (2.9)	1101 (1.2)	1113 (1.2)	1101 (1.3)	1031 (1.4)
Preexisting hypertension	310 054 (26.7)	10 242 (18.8)	23 948 (24.9)	27 794 (29.3)	26 953 (30.1)	27 114 (33.2)	27 119 (36.8)
Congenital heart disease	10 250 (0.9)	395 (0.7)	875 (0.9)	728 (0.8)	837 (0.9)	846 (1.0)	852 (1.2)
Systemic lupus erythematosus	15 092 (1.3)	498 (0.9)	1283 (1.3)	1172 (1.2)	1228 (1.4)	1292 (1.6)	1213 (1.6)
HIV	9612 (0.8)	487 (0.9)	695 (0.7)	762 (0.8)	667 (0.7)	687 (0.8)	584 (0.8)
Multiple gestation	214 087 (18.4)	10 360 (19.0)	18 208 (19.0)	17 476 (18.4)	15 604 (17.4)	13 606 (16.7)	12 751 (17.3)
Drug abuse	250 039 (21.5)	6353 (11.7)	18 887 (19.7)	21 921 (23.1)	25 035 (28.0)	25 489 (31.2)	24 114 (32.7)
Alcohol abuse	10 591 (0.9)	501 (0.9)	1066 (1.1)	731 (0.8)	649 (0.7)	622 (0.8)	634 (0.9)
Tobacco use	676 360 (58.2)	31 371 (57.5)	61 885 (64.5)	52 893 (55.8)	49 195 (55.0)	43 355 (53.1)	36 879 (50.0)
Cardiac valvular disease	24 109 (2.1)	2697 (4.9)	1974 (2.1)	1336 (1.4)	1167 (1.3)	1129 (1.4)	1060 (1.4)
Chronic congestive heart failure	10 250 (0.9)	395 (0.7)	875 (0.9)	728 (0.8)	837 (0.9)	846 (1)	852 (1.2)
Asthma	532 746 (45.8)	17 054 (31.3)	41 758 (43.5)	44 603 (47.1)	49 168 (54.9)	49 847 (61.1)	48 420 (65.7)
Preexisting diabetes	116 122 (10.0)	4688 (8.6)	10 306 (10.7)	9512 (10.0)	8702 (9.7)	8650 (10.6)	7241 (9.8)
Gestational diabetes	864 546 (74.3)	29 409 (53.9)	66 520 (69.3)	71 338 (75.3)	77 422 (86.5)	80 041 (98.1)	76 955 (104.4)
Obesity	1 057 844 (91.0)	18 550 (34.0)	76 178 (79.4)	91 702 (96.7)	116 107 (129.7)	122 908 (150.6)	121 771 (165.2)
Cystic fibrosis	959 (0.1)	36 (0.1)	55 (0.1)	92 (0.1)	54 (0.1)	130 (0.2)	126 (0.2)
Previous cesarean delivery	2 027 278 (174.3)	86 087 (157.9)	170 848 (178.0)	168 750 (178.0)	160 945 (179.8)	145 359 (178.1)	131 693 (178.6)
Other clinical conditions							
Hemorrhage	551 647 (47.4)	18 108 (33.2)	38 522 (40.1)	50 010 (52.8)	52 302 (58.4)	50 408 (61.8)	49 398 (67.0)
**Severe maternal morbidities, No. (rate per 10 000 discharges)**							
Acute myocardial infarction	370 (0.3)	7 (0.1)	18 (0.2)	24 (0.3)	40 (0.4)	54 (0.7)	40 (0.5)
Acute kidney failure	11 304 (9.7)	262 (4.8)	702 (7.3)	905 (9.5)	1275 (14.2)	1350 (16.5)	1426 (19.3)
Acute respiratory distress syndrome	11 430 (9.8)	245 (4.5)	820 (8.5)	899 (9.5)	994 (11.1)	1272 (15.6)	1695 (23.0)
Amniotic fluid embolism	571 (0.5)	25 (0.5)	36 (0.4)	51 (0.5)	55 (0.6)	44 (0.5)	37 (0.5)
Aneurysm	289 (0.2)	5 (0.1)	20 (0.2)	30 (0.3)	30 (0.3)	31 (0.4)	21 (0.3)
Cardiac arrest or ventricular fibrillation	1065 (0.9)	38 (0.7)	87 (0.9)	102 (1.1)	70 (0.8)	86 (1.1)	137 (1.9)
Cardioversion	1040 (0.9)	35 (0.6)	73 (0.8)	78 (0.8)	85 (0.9)	94 (1.2)	103 (1.4)
Disseminated intravascular coagulation	28 722 (24.7)	1418 (26.0)	2709 (28.2)	1848 (19.5)	1789 (20)	1594 (19.5)	1614 (21.9)
Eclampsia	8367 (7.2)	448 (8.2)	571 (5.9)	969 (10.2)	602 (6.7)	562 (6.9)	530 (7.2)
Heart failure or arrest during procedure	677 (0.6)	84 (1.5)	72 (0.8)	7 (0.1)	13 (0.1)	7 (0.1)	12 (0.2)
Puerperal cerebrovascular disorders	3492 (3.0)	186 (3.4)	260 (2.7)	287 (3.0)	274 (3.1)	268 (3.3)	291 (3.9)
Acute heart failure or pulmonary edema	6567 (5.6)	264 (4.8)	426 (4.4)	575 (6.1)	601 (6.7)	580 (7.1)	576 (7.8)
Severe anesthesia complications	1111 (1.0)	103 (1.9)	107 (1.1)	71 (0.7)	59 (0.7)	40 (0.5)	42 (0.6)
Sepsis	8594 (7.4)	249 (4.6)	606 (6.3)	760 (8.0)	858 (9.6)	871 (10.7)	973 (13.2)
Shock	7112 (6.1)	161 (3.0)	488 (5.1)	633 (6.7)	745 (8.3)	726 (8.9)	784 (10.6)
Sickle cell anemia with crisis	1131 (1.0)	56 (1.0)	82 (0.9)	86 (0.9)	99 (1.1)	88 (1.1)	68 (0.9)
Air and thrombotic embolism	2877 (2.5)	90 (1.7)	179 (1.9)	271 (2.9)	291 (3.3)	258 (3.2)	300 (4.1)
Blood transfusion	125 999 (108.4)	4746 (87.0)	10 498 (109.4)	9347 (98.6)	9971 (111.4)	9704 (118.9)	9727 (131.9)
Hysterectomy	12 738 (11.0)	476 (8.7)	977 (10.2)	1033 (10.9)	1121 (12.5)	1134 (13.9)	944 (12.8)
Temporary tracheostomy	324 (0.3)	14 (0.3)	26 (0.3)	15 (0.2)	17 (0.2)	27 (0.3)	57 (0.8)
Ventilation	4154 (3.6)	39 (0.7)	128 (1.3)	524 (5.5)	506 (5.7)	519 (6.4)	675 (9.2)
Any severe maternal morbidity	189 908 (163.3)	7374 (135.2)	15 366 (160.1)	14 410 (152)	15 250 (170.4)	14 961 (183.3)	15 192 (206.1)

### Maternal Comorbid Conditions

As shown in Table 1, obesity (91.0 per 1000 discharges), gestational diabetes (74.3 per 1000 discharges), and tobacco use (58.2 per 1000 discharges) were the most common comorbidities, followed by gestational hypertension, asthma, preeclampsia, preexisting hypertension, and substance use disorder. Compared with the prevalence in 2008, higher prevalence of sickle cell disease, gestational hypertension, severe preeclampsia, preexisting hypertension, substance use disorder, asthma, gestational diabetes, obesity, and hemorrhage were observed in 2021 (Table 1).

### Prevalence and Trend of SMMs

The unadjusted prevalence of any SMM was estimated to be 163.3 per 10 000 discharges for the overall sample from 2008 to 2021, with higher prevalence observed in 2021 (206.1 per 10 000 discharges) compared with 2008 (135.2 per 10 000 discharges). Blood transfusion was the most common SMM observed, with a prevalence of 108.4 per 10 000 discharges. Other relatively common SMMs included disseminated intravascular coagulation (24.7 per 10 000 discharges), hysterectomy (11.0 per 10 000 discharges), acute respiratory distress syndrome (9.8 per 10 000 discharges), acute kidney failure (9.7 per 10 000 discharges), sepsis (7.4 per 10 000 discharges), eclampsia (7.2 per 10 000 discharges), shock (6.1 per 10 000 discharges), and acute heart failure or pulmonary edema (5.6 per 10 000 discharges). Prevalence of acute kidney failure, acute respiratory distress syndrome, sepsis, shock, mechanical ventilation, blood transfusion, and hysterectomy were higher in 2021 than in 2008 (Table 1).

**Figure 1.**
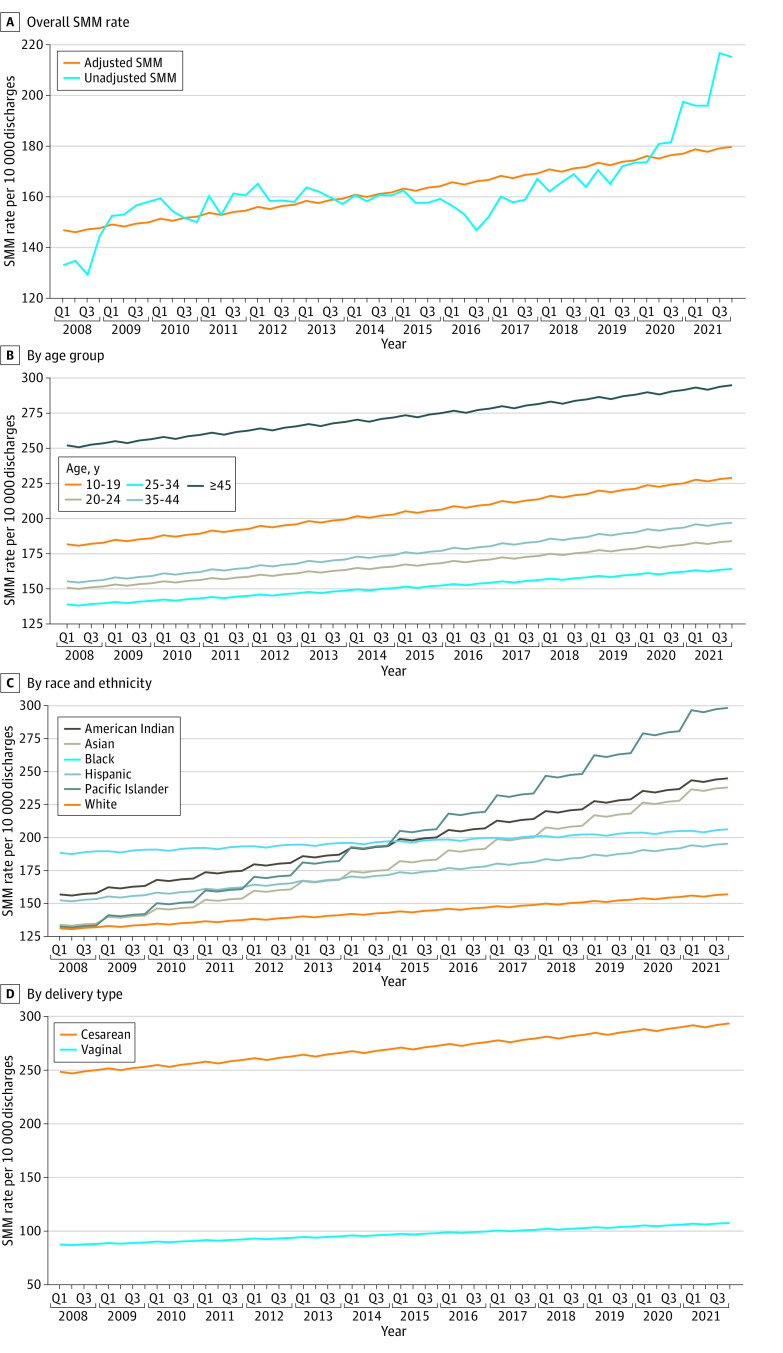
Trend of Unadjusted and Regression-Adjusted Severe Maternal Morbidity (SMM) Rates Among Hospital Inpatient Discharges for Newborn Delivery, 2008 to 2021, Overall and by Age Group, Race and Ethnicity, and Delivery Type Q indicates quarter.

As seen in Figure 1A, the adjusted prevalence of any SMM increased from Q1 2008 (146.8 per 10 000 discharges) to Q4 2021 (179.8 per 10 000 discharges). The increasing trend was observed in all age groups with the greatest change observed in patients aged 45 years or older and those aged 10 to 19 years (Figure 1B). Consistent increasing trend was also observed in all racial and ethnic groups, with the biggest increase observed among Pacific Islander patients (from 132.0 per 10 000 discharges in Q1 2008 to 298.8 per 10 000 discharges in Q4 2021), American Indian patients (from 156.5 per 10 000 discharges in Q1 2008 to 245.0 per 10 000 discharges in Q4 2021), and Asian patients (from 133.4 per 10 000 discharges in Q1 2008 to 238.2 per 10 000 discharges in Q4 2021) (Figure 1C). A significant increase in adjusted SMM prevalence was observed in patients undergoing cesarean delivery (from 252.4 per 10 000 discharges in Q1 of 2008 to 312.1 per 10 000 discharges in Q4 of 2021), and a similarly increasing trend was seen in patients with vaginal delivery (from 84.4 per 10 000 discharges in Q1 of 2008 to 108.4 per 10 000 discharges in Q4 of 2021) (Figure 1D).

### Unadjusted and Adjusted Trend of In-Hospital Delivery-Related Mortality

**Figure 2.**
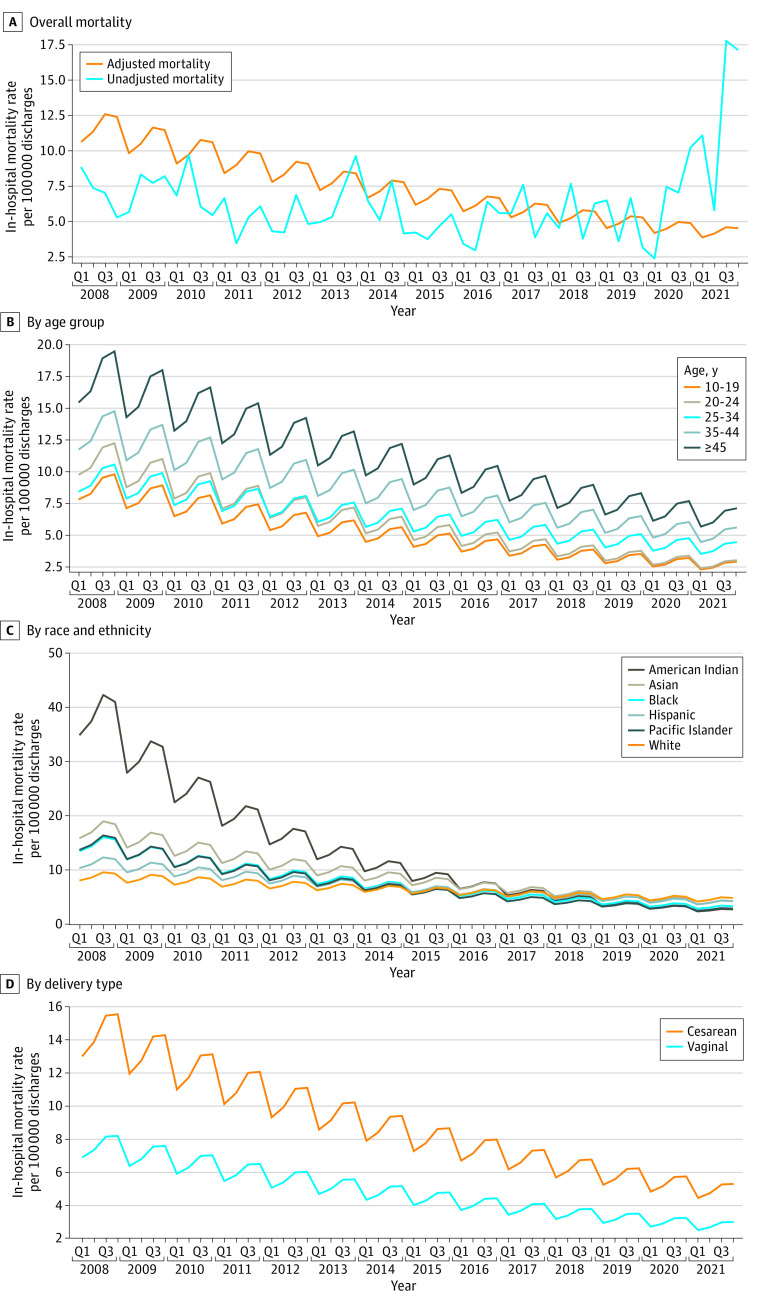
Trend of Unadjusted and Regression-Adjusted In-Hospital Mortality Among Hospital Inpatient Discharges for Newborn Delivery, 2008 to 2021, Overall and by Age Group, Race and Ethnicity, and Delivery Type Q indicates quarter.

As shown in Figure 2A, a downward trend was observed for in-hospital mortality among deliveries after adjusting for changes in patient demographic, visit, hospital, and clinical characteristics. From Q1 of 2008 to Q4 of 2021, the adjusted in-hospital mortality decreased from 10.6 per 100 000 discharges to 4.6 per 100 000 discharges. Each subsequent year after 2008 had an 11% decrease in odds of death compared with the previous year (adjusted odds ratio [aOR], 0.89; 95% CI, 0.87-0.92) (Table 2). There was an increase in mortality from Q2 of 2020 through Q4 of 2021 that may be associated with the COVID-19 pandemic. However, after controlling for COVID-19 diagnosis, the adjusted trend decreased consistently across the full study period. The downward trend for in-hospital mortality was observed in all age groups, with the biggest decrease occurring in patients aged 45 years or older (Figure 2B). A decreasing trend for in-hospital mortality was observed in all racial and ethnic groups. In particular, the greatest decrease in adjusted mortality was observed for American Indian patients: from 34.8 per 100 000 discharges in Q1 of 2008 to 2.7 per 100 000 discharges in Q4 of 2021 (Figure 2C; the 95% CI for mortality among American Indian patients is provided in eTable 4 in Supplement 1). In-hospital mortality consistently decreased during the study period for patients with cesarean delivery (from 12.6 per 100 000 discharges in Q1 of 2008 to 5.2 per 100 000 discharges in Q4 of 2021) and also for patients with vaginal delivery (from 6.6 per 100 000 discharges in Q1 of 2008 to 3.0 per 100 000 discharges in Q4 of 2021) (Figure 2D).

**Table 2.** Estimates From the Multivariable Logistic Regression of In-Hospital Mortality and Any SMM Among Hospital Inpatient Discharges for Newborn Delivery, 2008 to 2021

Variable	Adjusted OR (95% CI)		
	In-hospital mortality^a^	In-hospital mortality sensitivity analysis^b^	Any SMM^c^
Age group, y			
10-19	0.74 (0.48-1.14)	0.61 (0.42-0.89)	1.39 (1.36-1.42)
20-24	0.90 (0.70-1.15)	0.70 (0.56-0.86)	1.10 (1.08-1.11)
25-34	1 [Reference]	1 [Reference]	1 [Reference]
35-44	1.49 (1.22-1.84)	2.16 (1.83-2.54)	1.17 (1.16-1.19)
≥45	1.95 (0.83-4.55)	6.01 (3.16-11.41)	1.76 (1.65-1.88)
Year	0.89 (0.87-0.92)	0.95 (0.93-0.97)	1.02 (1.01-1.02)
Diagnosis code version (*ICD-10-CM* vs *ICD-9*-*CM*)	NE	NE	0.92 (0.90-0.94)
Race			
White	1 [Reference]	1 [Reference]	1 [Reference]
American Indian	1.86 (0.95-3.64)	1.93 (1.10-3.39)	1.41 (1.35-1.48)
Asian	1.37 (0.88-2.14)	1.61 (1.11-2.34)	1.33 (1.30-1.36)
Black	1.09 (0.86-1.37)	1.78 (1.47-2.15)	1.39 (1.38-1.41)
Hispanic	1.06 (0.81-1.39)	1.04 (0.83-1.31)	1.22 (1.20-1.24)
Other or unknown	1.02 (0.77-1.35)	1.19 (0.94-1.51)	1.21 (1.19-1.22)
Pacific Islander	0.89 (0.29-2.76)	1.25 (0.48-3.26)	1.53 (1.45-1.61)
Quarter			
First	0.80 (0.62-1.04)	0.91 (0.74-1.13)	0.99 (0.98-1.01)
Second	0.88 (0.68-1.14)	0.91 (0.74-1.13)	0.99 (0.98-1.00)
Third	1.02 (0.80-1.31)	1.03 (0.85-1.26)	1.00 (0.98-1.01)
Fourth	1 [Reference]	1 [Reference]	1 [Reference]
Urbanicity of hospital population served (rural vs urban)	NE	NE	0.88 (0.87-0.90)
Primary payer			
Commercial	1 [Reference]	1 [Reference]	1 [Reference]
Charity or indigent	1.51 (0.27-8.53)	2.36 (0.78-7.09)	1.57 (1.42-1.73)
Medicaid	1.73 (1.41-2.13)	1.66 (1.4-1.97)	1.22 (1.20-1.23)
Other	1.72 (1.25-2.36)	1.69 (1.31-2.20)	1.23 (1.21-1.25)
Teaching hospital (vs nonteaching)	NE	NE	1.18 (1.17-1.20)
Hospital region			
South Atlantic	1 [Reference]	1 [Reference]	1 [Reference]
East North Central	0.91 (0.67-1.24)	0.87 (0.68-1.11)	0.94 (0.93-0.95)
East South Central	1.42 (1.00-2.02)	1.29 (0.96-1.72)	0.90 (0.88-0.92)
Middle Atlantic	0.93 (0.68-1.27)	0.91 (0.70-1.18)	1.15 (1.13-1.16)
Mountain	1.04 (0.70-1.54)	1.31 (0.95-1.79)	0.87 (0.85-0.89)
New England	0.73 (0.35-1.53)	0.62 (0.32-1.18)	1.09 (1.06-1.12)
Pacific	0.66 (0.48-0.92)	0.66 (0.50-0.88)	0.84 (0.83-0.86)
West North Central	1.36 (0.90-2.07)	1.28 (0.91-1.80)	0.88 (0.86-0.90)
West South Central	1.07 (0.80-1.45)	1.11 (0.88-1.42)	1.09 (1.08-1.11)
Admission type			
Elective	1 [Reference]	1 [Reference]	1 [Reference]
Emergent	3.17 (2.48-4.04)	5.17 (4.26-6.26)	1.50 (1.48-1.52)
Urgent	1.20 (0.96-1.49)	1.55 (1.29-1.87)	1.08 (1.06-1.09)
Trauma center	16.65 (9.89-28.04)	16.21 (10.36-25.35)	1.40 (1.31-1.50)
Other or unknown	1.46 (0.90-2.36)	1.43 (0.96-2.15)	1.28 (1.25-1.31)
Delivery type			
Vaginal	1 [Reference]	1 [Reference]	1 [Reference]
Cesarean	2.28 (1.87-2.79)	3.94 (3.34-4.64)	3.00 (2.97-3.03)
Comorbidities (yes vs no)			
COVID-19 diagnosis	13.31 (8.95-19.79)	41.63 (31.7-54.67)	4.44 (4.23-4.66)
Placenta previa	NE	NE	6.32 (6.17-6.47)
Sickle cell disease	NE	NE	2.93 (2.82-3.05)
Gestational hypertension	NE	NE	1.29 (1.26-1.31)
Mild or unspecified preeclampsia	NE	NE	1.93 (1.89-1.97)
Severe preeclampsia	NE	NE	4.55 (4.48-4.62)
Chronic kidney disease	NE	NE	5.21 (4.99-5.44)
Preexisting hypertension	NE	NE	1.54 (1.50-1.57)
Chronic ischemic heart disease	NE	NE	3.92 (3.50-4.39)
Congenital heart disease	0.07 (0.01-0.34)	1.8 (0.46-7.08)	2.43 (2.23-2.66)
Systemic lupus erythematosus	3.68 (1.70-7.97)	7.63 (4.28-13.62)	1.95 (1.81-2.10)
HIV	3.94 (1.30-11.92)	3.03 (1.29-7.13)	1.47 (1.33-1.63)
Multiple gestation	NE	NE	2.20 (2.16-2.24)
Substance use disorder	1.38 (1.00-1.91)	3.44 (2.67-4.43)	1.71 (1.67-1.75)
Alcohol abuse	NE	NE	1.68 (1.52-1.85)
Cardiac valvular disease	0.19 (0.10-0.33)	6.73 (4.07-11.14)	3.50 (3.32-3.69)
Asthma	NE	NE	1.26 (1.24-1.28)
Preexisting diabetes	1.70 (1.20-2.42)	3.69 (2.81-4.86)	1.22 (1.18-1.25)
Gestational diabetes	NE	NE	0.96 (0.95-0.98)
Cystic fibrosis	40.17 (7.22-223.56)	35.97 (9.32-138.81)	1.76 (1.22-2.52)
Previous cesarean delivery	NE	NE	0.88 (0.87-0.89)
SMM (yes vs no)			
Acute kidney failure	1.30 (1.01-1.67)	Not included	Not included
Amniotic fluid embolism	7.67 (5.28-11.14)	Not included	Not included
Aneurysm	25.07 (7.40-84.91)	Not included	Not included
Disseminated intravascular coagulation	2.21 (1.69-2.88)	Not included	Not included
Puerperal cerebrovascular disorder	9.09 (6.11-13.51)	Not included	Not included
Sepsis	1.90 (1.45-2.51)	Not included	Not included
Shock	3.81 (2.96-4.91)	Not included	Not included
Air and thrombotic embolism	2.63 (1.79-3.86)	Not included	Not included
Sickle cell anemia with crisis	4.96 (2.17-11.33)	Not included	Not included
Bleeding complications (hemorrhage with no blood transfusion vs no bleeding)	2.73 (2.06-3.61)	Not included	Not included
Bleeding complications (blood transfusion vs no bleeding)	3.87 (3.05-4.91)	Not included	Not included
Hysterectomy	1.69 (1.24-2.318)	Not included	Not included
Respiratory complications^d^	5.92 (4.56-7.69)	Not included	Not included
Cardiovascular complications^e^	111.11 (86.69-142.42)	Not included	Not included

Abbreviations: *ICD-9-CM*, *International Classification of Diseases, Ninth Revision, Clinical Modification*; *ICD-10-CM*, *International Statistical Classification of Diseases, Tenth Revision, Clinical Modification*; NE, not entered in final model; OR, odds ratio; SMM, severe maternal morbidity.

^a^ Overall, 728 deaths among 11 628 380 inpatient hospitalizations were included in the analysis; 58 patients reported by hospitals with in-hospital mortality followed by readmission were excluded from the regression of mortality. Additional covariates included in the regression of mortality are described in the Statistical Analysis section.

^b^ A logistic regression of mortality without SMMs as covariates was performed as a sensitivity analysis.

^c^ A total of 189 908 discharges with SMM (of 11 628 438 inpatient hospitalizations) were included in the analysis. Additional covariates included in the regression of severe maternal morbidity are described in the Statistical Analysis section.

^d^ Respiratory complications included acute respiratory distress syndrome, temporary tracheostomy and ventilation as defined by the US Centers for Disease Control and Prevention.

^e^ Cardiovascular complications included any of the following severe maternal morbidities: acute myocardial infarction, cardiac arrest or ventricular fibrillation, conversion of cardiac rhythm, heart failure or arrest during surgery or procedure, and pulmonary edema or acute heart failure as defined by the US Centers for Disease Control and Prevention.

### Risk Factors for In-Hospital Mortality and SMM

Compared with patients aged 25 to 34 years, those between 35 and 44 years had higher odds of dying during the index hospitalization (aOR, 1.49; 95% CI, 1.22-1.84). Although the association between race and mortality was not statistically significant in the regression in which SMMs were included as covariates, a sensitivity analysis showed that American Indian (aOR, 1.93, 95% CI, 1.10-3.39), Black (aOR, 1.78; 95% CI, 1.47-2.15), and Asian patients (aOR, 1.61, 95% CI, 1.11-2.34) had increased risk of death compared with White patients, suggesting that the racial disparity was partly attributable to the difference in the SMM rates across racial and ethnic groups. The mortality of Pacific Islander patients and Hispanic patients was not statistically significantly different from White patients in the sensitivity analysis. Patients with cesarean delivery had 2-fold higher odds of death than those with vaginal delivery (aOR, 2.28; 95% CI, 1.87-2.79). Patients with a COVID-19 diagnosis had a 13-fold increased odds of mortality compared with those without COVID-19 (aOR, 13.31; 95% CI, 8.95-19.79). Among comorbidity and acute complications assessed, cardiac complications, cystic fibrosis, aneurysm, trauma, and puerperal cerebrovascular disorder were among the risk factors associated with death during delivery-related hospitalization (Table 2).

As seen in Table 2, after adjusting for other risk factors and compared with patients aged 25 to 34 years, both patients younger than 24 years (eg, age 10-19 years: aOR, 1.39; 95% CI, 1.36-1.42) and older than 35 years (eg, age ≥45 years: aOR, 1.76; 95% CI, 1.65-1.88) had increased odds of SMM. All minority racial and ethnic groups were associated with increased odds of experiencing any SMM (Pacific Islander: aOR, 1.53; 95% CI, 1.45-1.61; American Indian: aOR, 1.41; 95% CI, 1.35-1.48; Black: aOR, 1.39; 95% CI, 1.38-1.41; Asian: aOR, 1.33; 95% CI, 1.30-1.36; Hispanic: aOR, 1.22; 95% CI, 1.20-1.24). Cesarean delivery (aOR, 3.00; 95% CI, 2.97- 3.03) and COVID-19 diagnosis (aOR, 4.44; 95% CI, 4.23-4.66) were also associated with substantially higher adjusted odds of SMM. Among all the chronic comorbidities assessed, placenta previa (aOR, 6.32; 95% CI, 6.17-6.47), chronic kidney disease (aOR, 5.21; 95% CI, 4.99-5.44), severe preeclampsia (aOR, 4.55; 95% CI, 4.48-4.62), cardiac valvular disease (aOR, 3.50; 95% CI, 3.32-3.69), chronic ischemic heart disease (aOR, 3.92; 95% CI, 3.50-4.39), and sickle cell disease (aOR, 2.93; 95% CI, 2.82-3.05) were associated with the highest odds of experiencing SMM (Table 2).

## Discussion

This cross-sectional study examined rates of delivery-related in-hospital maternal mortality and SMM in a large national inpatient database. In this sample encompassing more than 11 million inpatient discharges, delivery-related in-hospital mortality was found to decrease significantly over a period of 14 years. The adjusted mortality per 100 000 discharges decreased by more than 50% from Q1 of 2008 to Q4 of 2021, likely demonstrating the impact of national strategies focused on improving the maternal quality of care provided by the hospitals during delivery-related hospitalizations. In contrast, the rates of overall SMM increased over time for the overall population, which may be attributable to preexisting conditions and the increasing trend in the age of delivering patients in the past decade. The increasing trend of adjusted SMM rates was seen in all racial and ethnic minority groups and was most prominent in Asian, American Indian, and Pacific Islander patients. The fact that many of the comorbid conditions are risk factors for mortality and SMM indicates that it is essential to consider comorbid conditions when assessing SMM and mortality and that better management of patients’ comorbid conditions during pregnancy may help reduce SMM occurrence and ultimately decrease mortality risk. Further improvement in patient outcomes could be achieved if patients with known risk factors could access improved care during pregnancy and during hospital delivery.

Delivery-related in-hospital maternal mortality in this study was lower than that reported in PMSS data, which defined pregnancy-related death as death during pregnancy or within 1 year of the end of pregnancy, from a cause related to pregnancy or its management.^3^ PMSS data showed an increasing trend in pregnancy-related mortality during 1987 to 2017, which differs from our findings. A plausible explanation for these differences is that the timeframe for assessing mortality was substantially different between our study and the PMSS. Our study focused on mortality during delivery-related hospitalizations, which was associated with the change in quality of care for all patients in a hospital setting. In contrast, the PMSS estimates cover the entire pregnancy and postpartum period, which are associated with the overall burden of deaths among pregnant patients. Because a proportion of pregnancy-related deaths occur during delivery hospitalization, the differences between our findings and the PMSS estimates reinforced the importance of examining mortality separately for different stages of pregnancy and postpartum.

The study found that mortality risk was associated with several factors, including advanced maternal age, Medicaid as primary insurance, cesarean delivery, comorbid conditions, and severe complications during delivery. Similarly, a maternal age younger than 19 years or older than 35 years; being Asian, American Indian, Black, Hispanic, Pacific Islander; cesarean delivery; Medicaid enrollment; and maternal comorbid conditions were associated with higher risk of developing SMM during delivery. The racial and ethnic differences observed in delivery-related maternal mortality seem to be at least partly attributable to the racial and ethnic variation observed in SMM based on the main and sensitivity analyses of this study. Therefore, further research and understanding on the causes of both mortality and SMM, including the impact of comorbidities on maternal outcomes, is needed. Additionally, developing a national hospital measure to more clearly identify and reduce SMMs will likely have a beneficial impact on improving national quality strategies aimed at improving maternal health outcomes in the United States.

### Limitations

The study has limitations. The PHD is a hospital administrative database and does not include as much clinical details as electronic health records. Identifying clinical conditions and procedures relied on the accuracy of hospital-reported diagnosis and procedure codes. Coding errors may lead to misclassification of variables. The definition of mortality was based on in-hospital death during the visit for delivery, without accounting for death before delivery admission or after discharge.

Maternal comorbid conditions were defined based on diagnosis during the visit for delivery. Conditions occurring before admission may not have been captured. Since the study spanned 14 years, there were changes in how hospitals collected and reported race and ethnicity. Hispanic was reported as a race category before 2011, while ethnicity was listed as a separate field in the patient admission form in later years. Hispanic race or ethnicity as defined in this study included patients who reported Hispanic as their race before 2011 and those who reported Hispanic as their ethnicity in 2012 and later, regardless of their reported race.

## Conclusions

This large national study found a decreasing trend of in-hospital delivery-related maternal mortality during 2008 to 2021, regardless of racial or ethnic group, age, or mode of delivery, likely demonstrating the impact of national and local strategies focused on improving the maternal quality of care provided by hospitals during delivery-related hospitalizations. Risk factors for in-hospital delivery-related mortality included cesarean delivery, COVID-19 diagnosis, and comorbidities and acute complications. Analysis indicated that American Indian, Black, and Asian patients had a statistically significant increased risk of death compared with White patients only when not controlling for SMMs, suggesting that the racial difference in mortality could be at least partly attributable to the differences in SMM rates across racial groups (analysis of Pacific Islander and Hispanic patients were not statistically significant).

From 2008 to 2021, there was an increasing trend of SMM rates, and chronic comorbid conditions were associated with higher rates. SMMs are known risk factors of maternal deaths and impose substantial social and economic burdens. Notably, disparities in both mortality and SMM remained across age, delivery mode, and racial and ethnic groups. These characteristics should be considered when designing maternal care quality improvement programs. As current national strategies increasingly focus on improving delivery-related maternal outcomes among high-risk groups, including racial and ethnic minority groups, it will become important to evaluate the effectiveness of these strategies in reducing occurrences of maternal mortality and SMM.

## Notes

Supplement 1. **eTable 1.** *ICD-9-CM* and *ICD-10-CM* Diagnosis and Procedure Codes Used for Patient Inclusion and Exclusion **eTable 2.** *ICD-9-CM* and *ICD-10-CM* Diagnosis and Procedure Codes Used to Identify Severe Maternal Morbidities **eTable 3.** *ICD-9-CM* and *ICD-10-CM* Diagnosis Codes Used to Identify Maternal Comorbidities **eTable 4.** Rates of Adjusted Mortality for American Indian Patients with 95% Poisson CIs

Supplement 2.
Data Sharing Statement
