# Supplementary material for: Trends in Maternal Mortality and Severe Maternal Morbidity During Delivery-Related Hospitalizations in the United States, 2008 to 2021
Source: JAMA Netw Open. 2023 Jun 22;6(6):e2317641. doi: 10.1001/jamanetworkopen.2023.17641 (PMC10288331; doi:10.1001/jamanetworkopen.2023.17641)
Supplement: Supplement 1. — eTable 1. ICD-9-CM and ICD-10-CM Diagnosis and Procedure Codes Used for Patient Inclusion and Exclusion eTable 2. ICD-9-CM and ICD-10-CM Diagnosis and Procedure Codes Used to Identify Severe Maternal Morbidities eTable 3. ICD-9-CM and ICD-10-CM Diagnosis Codes Used to Identify Maternal Comorbidities eTable 4. Rates of Adjusted Mortality for American Indian Patients with 95% Poisson CIs [file jamanetwopen-e2317641-s001.pdf]

Supplemental Online Content

Fink DA, Kilday D, Cao Z, et al. Trends in maternal mortality and severe maternal morbidity during delivery-related hospitalizations in the United States, 2008 to 2021. *JAMA Netw Open.* 2023;6(6):e2317641. doi:10.1001/jamanetworkopen.2023.17641

eTable 1. *ICD-9-CM* and *ICD-10-CM* Diagnosis and Procedure Codes Used for Patient Inclusion and Exclusion

eTable 2. *ICD-9-CM* and *ICD-10-CM* Diagnosis and Procedure Codes Used to Identify Severe Maternal Morbidities

eTable 3. *ICD-9-CM* and *ICD-10-CM* Diagnosis Codes Used to Identify Maternal Comorbidities

eTable 4. Rates of Adjusted Mortality for American Indian Patients with 95% Poisson CIs

This supplemental material has been provided by the authors to give readers additional information about their work.

© 2023 Fink DA et al. *JAMA Network Open.*

eTable 1. *ICD-9-CM* and *ICD-10-CM* Diagnosis and Procedure Codes Used for Patient Inclusion and Exclusion

	ICD-9-CM	Descriptions	ICD-10-CM	Descriptions
**Delivery Diagnosis**				
	V27.0	Outcome of delivery, single liveborn	Z37.0	Single live birth
	V27.1	Outcome of delivery, single stillborn	Z37.1	Single stillbirth
	V27.2	Outcome of delivery, twins, both liveborn	Z37.2	Twins, both liveborn
	V27.3	Outcome of delivery, twins, one liveborn and one stillborn	Z37.3	Twins, one liveborn and one stillborn
	V27.4	Outcome of delivery, twins, both stillborn	Z37.4	Twins, both stillborn
	V27.5	Outcome of delivery, other multiple birth, all liveborn	Z37.50	Multiple births, unspecified, all liveborn
	V27.6	Outcome of delivery, other multiple birth, some liveborn	Z37.51	Triplets, all liveborn
	V27.7	Outcome of delivery, other multiple birth, all stillborn	Z37.52	Quadruplets, all liveborn
	V27.9	Outcome of delivery, unspecified outcome of delivery	Z37.53	Quintuplets, all liveborn
	669.70	Cesarean delivery, without mention of indication, unspecified as to episode of care or not applicable	Z37.54	Sextuplets, all liveborn
	669.71	Cesarean delivery, without mention of indication, delivered, with or without mention of antepartum condition	Z37.59	Other multiple births, all liveborn
			Z37.60	Multiple births, unspecified, some liveborn
			Z37.61	Triplets, some liveborn
			Z37.62	Quadruplets, some liveborn
			Z37.63	Quintuplets, some liveborn
			Z37.64	Sextuplets, some liveborn
			Z37.69	Other multiple births, some liveborn
			Z37.7	Other multiple births, all stillborn
			Z37.9	Outcome of delivery, unspecified
			O75.82	Onset (spontaneous) of labor after 37 completed weeks of gestation but before 39 completed weeks gestation, with delivery by (planned) cesarean section
			O80	Encounter for full-term uncomplicated delivery

© 2023 Fink DA et al. *JAMA Network Open.*

ICD-9-CM		Descriptions	ICD-10-CM	Descriptions
			O82	Encounter for cesarean delivery without indication
**Delivery Procedure**				
	72.0	Low forceps operation	10D00Z0	Extraction of Products of Conception, Classical, Open Approach
	72.1	Low forceps operation with episiotomy	10D00Z1	Extraction of Products of Conception, Low Cervical, Open Approach
	72.21	Mid forceps operation with episiotomy	10D00Z2	Extraction of Products of Conception, Extraperitoneal, Open Approach
	72.29	Other mid forceps operation	10D07Z3	Extraction of Products of Conception, Low Forceps, Via Natural or Artificial Opening
	72.31	High forceps operation with episiotomy	10D07Z4	Extraction of Products of Conception, Mid Forceps, Via Natural or Artificial Opening
	72.39	Other high forceps operation	10D07Z5	Extraction of Products of Conception, High Forceps, Via Natural or Artificial Opening
	72.4	Forceps rotation of fetal head	10D07Z6	Extraction of Products of Conception, Vacuum, Via Natural or Artificial Opening
	72.51	Partial breech extraction with forceps to aftercoming head	10D07Z7	Extraction of Products of Conception, Internal Version, Via Natural or Artificial Opening
	72.52	Other partial breech extraction	10D07Z8	Extraction of Products of Conception, Other, Via Natural or Artificial Opening
	72.53	Total breech extraction with forceps to aftercoming head	10E0XZZ	Delivery of Products of Conception, External Approach
	72.54	Other total breech extraction		
	72.6	Forceps application to aftercoming head		
	72.71	Vacuum extraction with episiotomy		
	72.79	Other vacuum extraction		
	72.8	Other specified instrumental delivery		
	72.9	Unspecified instrumental delivery		
	73.22	Internal and combined version with extraction		
	73.59	Other manually assisted delivery		
	73.6	Episiotomy		

© 2023 Fink DA et al. *JAMA Network Open.*

	ICD-9-CM	Descriptions	ICD-10-CM	Descriptions
	74.0	Classical cesarean section		
	74.1	Low cervical cesarean section		
	74.2	Extraperitoneal cesarean section		
	74.4	Cesarean section of other specified type		
	74.99	Other cesarean section of unspecified type		
**Delivery DRG**				
	765	Cesarean Section With CC/MCC	765	Cesarean section w cc/mcc
	766	Cesarean Section Without CC/MCC	766	Cesarean section w/o cc/mcc
	767	Vaginal Delivery With Sterilization And/Or D&C	767	Vaginal delivery w sterilization &/or d&c
	768	Vaginal Delivery With O.R. Procedure Except Sterilization And/Or D&C	768	Vaginal delivery w o.r. proc except steril &/or d&c
	774	Vaginal Delivery With Complicating Diagnoses	774	Vaginal delivery w complicating diagnoses
	775	Vaginal Delivery Without Complicating Diagnosis	775	Vaginal delivery w/o complicating diagnoses
			783	Cesarean section w sterilization w mcc
			784	Cesarean section w sterilization w cc
			785	Cesarean section w sterilization w/o cc/mcc
			786	Cesarean section w/o sterilization w mcc
			787	Cesarean section w/o sterilization w cc
			788	Cesarean section w/o sterilization w/o cc/mcc
			796	Vaginal delivery w sterilization/d&c w mcc
			797	Vaginal delivery w sterilization/d&c w cc
			798	Vaginal delivery w sterilization/d&c wo cc/mcc
			805	Vaginal delivery without sterilization/d&c with mcc
			806	Vaginal delivery without sterilization/d&c with cc
			807	Vaginal delivery without sterilization/d&c without cc/mcc
**Pregnancy with Abortive Outcome Diagnosis**				
	630	Hydatidiform mole	O00.0	Abdominal pregnancy
	631	Other abnormal product of conception	O00.1	Tubal pregnancy
	632	Missed abortion	O00.2	Ovarian pregnancy

© 2023 Fink DA et al. *JAMA Network Open.*

ICD-9-CM	Descriptions	ICD-10-CM	Descriptions
633	Ectopic pregnancy	O00.8	Other ectopic pregnancy
634	Spontaneous abortion	O00.9	Ectopic pregnancy, unspecified
635	Legally induced abortion	O01.0	Classical hydatidiform mole
636	Illegal abortion	O01.1	Incomplete and partial hydatidiform mole
637	Unspecified abortion	O01.9	Hydatidiform mole, unspecified
638	Failed attempted abortion	O02.0	Blighted ovum and nonhydatidiform mole
639	Complications following abortion and ectopic and molar pregnancies	O02.1	Missed abortion
		O02.81	Inappropriate change in quantitative human chorionic gonadotropin (hCG) in early pregnancy
		O02.89	Other abnormal products of conception
		O02.9	Abnormal product of conception, unspecified
		O03.0	Genital tract and pelvic infection following incomplete spontaneous abortion
		O03.1	Delayed or excessive hemorrhage following incomplete spontaneous abortion
		O03.2	Embolism following incomplete spontaneous abortion
		O03.30	Unspecified complication following incomplete spontaneous abortion
		O03.31	Shock following incomplete spontaneous abortion
		O03.32	Renal failure following incomplete spontaneous abortion
		O03.33	Metabolic disorder following incomplete spontaneous abortion
		O03.34	Damage to pelvic organs following incomplete spontaneous abortion
		O03.35	Other venous complications following incomplete spontaneous abortion
		O03.36	Cardiac arrest following incomplete spontaneous abortion

© 2023 Fink DA et al. *JAMA Network Open.*

ICD-9-CM	Descriptions	ICD-10-CM	Descriptions
		O03.37	Sepsis following incomplete spontaneous abortion
		O03.38	Urinary tract infection following incomplete spontaneous abortion
		O03.39	Incomplete spontaneous abortion with other complications
		O03.4	Incomplete spontaneous abortion without complication
		O03.5	Genital tract and pelvic infection following complete or unspecified spontaneous abortion
		O03.6	Delayed or excessive hemorrhage following complete or unspecified spontaneous abortion
		O03.7	Embolism following complete or unspecified spontaneous abortion
		O03.80	Unspecified complication following complete or unspecified spontaneous abortion
		O03.81	Shock following complete or unspecified spontaneous abortion
		O03.82	Renal failure following complete or unspecified spontaneous abortion
		O03.83	Metabolic disorder following complete or unspecified spontaneous abortion
		O03.84	Damage to pelvic organs following complete or unspecified spontaneous abortion
		O03.85	Other venous complications following complete or unspecified spontaneous abortion
		O03.86	Cardiac arrest following complete or unspecified spontaneous abortion
		O03.87	Sepsis following complete or unspecified spontaneous abortion

© 2023 Fink DA et al. *JAMA Network Open.*

ICD-9-CM	Descriptions	ICD-10-CM	Descriptions
		O03.88	Urinary tract infection following complete or unspecified spontaneous abortion
		O03.89	Complete or unspecified spontaneous abortion with other complications
		O03.9	Complete or unspecified spontaneous abortion without complication
		O04.5	Genital tract and pelvic infection following (induced) termination of pregnancy
		O04.6	Delayed or excessive hemorrhage following (induced) termination of pregnancy
		O04.7	Embolism following (induced) termination of pregnancy
		O04.80	(Induced) termination of pregnancy with unspecified complications
		O04.81	Shock following (induced) termination of pregnancy
		O04.82	Renal failure following (induced) termination of pregnancy
		O04.83	Metabolic disorder following (induced) termination of pregnancy
		O04.84	Damage to pelvic organs following (induced) termination of pregnancy
		O04.85	Other venous complications following (induced) termination of pregnancy
		O04.86	Cardiac arrest following (induced) termination of pregnancy
		O04.87	Sepsis following (induced) termination of pregnancy
		O04.88	Urinary tract infection following (induced) termination of pregnancy
		O04.89	(Induced) termination of pregnancy with other complications
		O07.0	Genital tract and pelvic infection following failed attempted termination of pregnancy

© 2023 Fink DA et al. *JAMA Network Open.*

ICD-9-CM	Descriptions	ICD-10-CM	Descriptions
		O07.1	Delayed or excessive hemorrhage following failed attempted termination of pregnancy
		O07.2	Embolism following failed attempted termination of pregnancy
		O07.30	Failed attempted termination of pregnancy with unspecified complications
		O07.31	Shock following failed attempted termination of pregnancy
		O07.32	Renal failure following failed attempted termination of pregnancy
		O07.33	Metabolic disorder following failed attempted termination of pregnancy
		O07.34	Damage to pelvic organs following failed attempted termination of pregnancy
		O07.35	Other venous complications following failed attempted termination of pregnancy
		O07.36	Cardiac arrest following failed attempted termination of pregnancy
		O07.37	Sepsis following failed attempted termination of pregnancy
		O07.38	Urinary tract infection following failed attempted termination of pregnancy
		O07.39	Failed attempted termination of pregnancy with other complications
		O07.4	Failed attempted termination of pregnancy without complication
		O08.0	Genital tract and pelvic infection following ectopic and molar pregnancy
		O08.1	Delayed or excessive hemorrhage following ectopic and molar pregnancy
		O08.2	Embolism following ectopic and molar pregnancy
		O08.3	Shock following ectopic and molar pregnancy

© 2023 Fink DA et al. *JAMA Network Open.*

ICD-9-CM	Descriptions	ICD-10-CM	Descriptions
		O08.4	Renal failure following ectopic and molar pregnancy
		O08.5	Metabolic disorders following an ectopic and molar pregnancy
		O08.6	Damage to pelvic organs and tissues following an ectopic and molar pregnancy
		O08.7	Other venous complications following an ectopic and molar pregnancy
		O08.81	Cardiac arrest following an ectopic and molar pregnancy
		O08.82	Sepsis following ectopic and molar pregnancy
		O08.83	Urinary tract infection following an ectopic and molar pregnancy
		O08.89	Other complications following an ectopic and molar pregnancy
		O08.9	Unspecified complication following an ectopic and molar pregnancy
**Pregnancy with Abortive Outcome Procedure**			
69.01	Dilation and curettage for termination of pregnancy	10A00ZZ	Abortion of Products of Conception, Open Approach
69.51	Aspiration curettage of uterus for termination of pregnancy	10A03ZZ	Abortion of Products of Conception, Percutaneous Approach
74.91	Hysterotomy to terminate pregnancy	10A04ZZ	Abortion of Products of Conception, Percutaneous Endoscopic Approach
75.0	Intra-amniotic injection for abortion	10A07Z6	Abortion of Products of Conception, Vacuum, Via Natural or Artificial Opening
		10A07ZW	Abortion of Products of Conception, Laminaria, Via Natural or Artificial Opening
		10A07ZX	Abortion of Products of Conception, Abortifacient, Via Natural or Artificial Opening
		10A07ZZ	Abortion of Products of Conception, Via Natural or Artificial Opening

© 2023 Fink DA et al. *JAMA Network Open.*

ICD-9-CM	Descriptions	ICD-10-CM	Descriptions
		10A08ZZ	Abortion of Products of Conception, Via Natural or Artificial Opening Endoscopic

© 2023 Fink DA et al. *JAMA Network Open.*

eTable 2. ***ICD-9-CM*** **and** ***ICD-10-CM*** **Diagnosis and Procedure Codes Used to Identify Severe Maternal Morbidities**

ICD-9-CM	Descriptions	ICD-10-CM	Descriptions
**Acute Myocardial Infarction Diagnosis**			
410.xx	Acute myocardial infarction	I21.01	ST elevation (STEMI) myocardial infarction involving left main coronary artery
		I21.02	ST elevation (STEMI) myocardial infarction involving left anterior descending coronary artery
		I21.09	ST elevation (STEMI) myocardial infarction involving other coronary artery of anterior wall
		I21.11	ST elevation (STEMI) myocardial infarction involving right coronary artery
		I21.19	ST elevation (STEMI) myocardial infarction involving other coronary artery of inferior wall
		I21.21	ST elevation (STEMI) myocardial infarction involving left circumflex coronary artery
		I21.29	ST elevation (STEMI) myocardial infarction involving other sites
		I21.3	ST elevation (STEMI) myocardial infarction of unspecified site
		I21.4	Non-ST elevation (NSTEMI) myocardial infarction
		I21.9	Acute myocardial infarction, unspecified
		I21.A1	Myocardial infarction type 2
		I21.A9	Other myocardial infarction type
		I22.0	Subsequent ST elevation (STEMI) myocardial infarction of anterior wall
		I22.1	Subsequent ST elevation (STEMI) myocardial infarction of inferior wall
		I22.2	Subsequent non-ST elevation (NSTEMI) myocardial infarction
		I22.8	Subsequent ST elevation (STEMI) myocardial infarction of other sites
		I22.9	Subsequent ST elevation (STEMI) myocardial infarction of unspecified site
**Acute Renal Failure Diagnosis**			
584.5	Acute kidney failure with	N17.0	Acute kidney failure with tubular necrosis

© 2023 Fink DA et al. *JAMA Network Open.*

	ICD-9-CM	Descriptions	ICD-10-CM	Descriptions
		lesion of tubular necrosis		
	584.6	Acute kidney failure with lesion of renal cortical necrosis	N17.1	Acute kidney failure with acute cortical necrosis
	584.7	Acute kidney failure with lesion of renal medullary [papillary] necrosis	N17.2	Acute kidney failure with medullary necrosis
	584.8	Acute kidney failure with other specified pathological lesion in kidney	N17.8	Other acute kidney failure
	584.9	Acute kidney failure, unspecified	N17.9	Acute kidney failure, unspecified
	669.3x	Acute kidney failure following labor and delivery	O90.4	Postpartum acute kidney failure
**Adult Respiratory Distress Syndrome Diagnosis**				
	518.5x	Pulmonary insufficiency following trauma and surgery	J80	Acute respiratory distress syndrome
	518.81	Acute respiratory failure	J95.1	Acute pulmonary insufficiency following thoracic surgery
	518.82	Other pulmonary insufficiency, not elsewhere classified	J95.2	Acute pulmonary insufficiency following nonthoracic surgery

© 2023 Fink DA et al. *JAMA Network Open.*

ICD-9-CM	Descriptions	ICD-10-CM	Descriptions
518.84	Acute and chronic respiratory failure	J95.3	Chronic pulmonary insufficiency following surgery
799.1	Respiratory arrest	J95.821	Acute postprocedural respiratory failure
		J95.822	Acute and chronic postprocedural respiratory failure
		J96.00	Acute respiratory failure unspecified whether with hypoxia or hypercapnia
		J96.01	Acute respiratory failure with hypoxia
		J96.02	Acute respiratory failure with hypercapnia
		J96.20	Acute and chronic respiratory failure unspecified whether with hypoxia or hypercapnia
		J96.21	Acute and chronic respiratory failure with hypoxia
		J96.22	Acute and chronic respiratory failure with hypercapnia
		R09.2	Respiratory arrest
**Amniotic Fluid Embolism Diagnosis**			
		O88.112	Amniotic fluid embolism in second trimester
		O88.113	Amniotic fluid embolism in third trimester
		O88.119	Amniotic fluid embolism in pregnancy
		O88.12	Amniotic fluid embolism in childbirth
		O88.13	Amniotic fluid embolism in puerperium
**Aneurysm Diagnosis**			
441.xx	Aortic aneurysm and dissection	I71.00	Dissection of unspecified site of aorta
		I71.01	Dissection of thoracic aorta
		I71.02	Dissection of abdominal aorta
		I71.03	Dissection of thoracoabdominal aorta
		I71.1	Thoracic aortic aneurysm, ruptured
		I71.2	Thoracic aortic aneurysm, without rupture
		I71.3	Abdominal aortic aneurysm, ruptured
		I71.4	Abdominal aortic aneurysm, without rupture
		I71.5	Thoracoabdominal aortic aneurysm, ruptured
		I71.6	Thoracoabdominal aortic aneurysm, without rupture

© 2023 Fink DA et al. *JAMA Network Open.*

ICD-9-CM	Descriptions	ICD-10-CM	Descriptions
		I71.8	Aortic aneurysm of unspecified site, ruptured
		I71.9	Aortic aneurysm of unspecified site, without rupture
		I79.0	Aneurysm of aorta in diseases classified elsewhere
**Cardiac arrest/ventricular fibrillation Diagnosis**			
427.41	Ventricular fibrillation	I49.01	Ventricular fibrillation
427.42	Ventricular flutter	I49.02	Ventricular flutter
427.5	Cardiac arrest	I46.2	Cardiac arrest due to underlying cardiac condition
		I46.8	Cardiac arrest due to other underlying condition
		I46.9	Cardiac arrest, cause unspecified
**Disseminated Intravascular Coagulation Diagnosis**			
286.6	Defibrination syndrome	D65	Disseminated intravascular coagulation [defibrination syndrome]
286.9	Other and unspecified coagulation defects	D68.8	Other specified coagulation defects
666.3x	Postpartum coagulation defects	D68.9	Coagulation defect, unspecified
		O72.3	Postpartum coagulation defects
**Eclampsia Diagnosis**			
642.6x	Eclampsia complicating pregnancy childbirth or the puerperium	O15.00	Eclampsia complicating pregnancy, unspecified trimester
		O15.02	Eclampsia in pregnancy, second trimester
		O15.03	Eclampsia in pregnancy, third trimester
		O15.1	Eclampsia in labor
		O15.2	Eclampsia in the puerperium
		O15.9	Eclampsia, unspecified as to time period
**Heart failure/arrest during procedure or surgery Diagnosis**			
997.1	Cardiac complications,	I97.120	Postprocedural cardiac arrest following cardiac surgery

© 2023 Fink DA et al. *JAMA Network Open.*

ICD-9-CM	Descriptions	ICD-10-CM	Descriptions
	not elsewhere classified		
		I97.121	Postprocedural cardiac arrest following other surgery
		I97.130	Postprocedural heart failure following cardiac surgery
		I97.131	Postprocedural heart failure following other surgery
		I97.710	Intraoperative cardiac arrest during cardiac surgery
		I97.711	Intraoperative cardiac arrest during other surgery
**Puerperal Cerebrovascular Disorder Diagnosis**			
430.xx	Subarachnoid hemorrhage	I60.00	Nontraumatic subarachnoid hemorrhage from unspecified carotid siphon and bifurcation
431.xx	Intracerebral hemorrhage	I60.01	Nontraumatic subarachnoid hemorrhage from right carotid siphon and bifurcation
432.xx	Other and unspecified intracranial hemorrhage	I60.02	Nontraumatic subarachnoid hemorrhage from left carotid siphon and bifurcation
433.xx	Occlusion and stenosis of precerebral arteries	I60.10	Nontraumatic subarachnoid hemorrhage from unspecified middle cerebral artery
434.xx	Occlusion of cerebral arteries	I60.11	Nontraumatic subarachnoid hemorrhage from right middle cerebral artery
436.xx	Acute, but ill-defined, cerebrovascular disease	I60.12	Nontraumatic subarachnoid hemorrhage from left middle cerebral artery
437.xx	Other and ill-defined cerebrovascular disease	I60.2	Nontraumatic subarachnoid hemorrhage from anterior communicating artery
671.5x	Other phlebitis and thrombosis in pregnancy and the puerperium	I60.20	Nontraumatic subarachnoid hemorrhage from unspecified anterior communicating artery

© 2023 Fink DA et al. *JAMA Network Open.*

	ICD-9-CM	Descriptions	ICD-10-CM	Descriptions
	674.0x	Cerebrovascular disorders in the puerperium	I60.21	Nontraumatic subarachnoid hemorrhage from right anterior communicating artery
	997.02	Iatrogenic cerebrovascular infarction or hemorrhage	I60.22	Nontraumatic subarachnoid hemorrhage from left anterior communicating artery
			I60.30	Nontraumatic subarachnoid hemorrhage from unspecified posterior communicating artery
			I60.31	Nontraumatic subarachnoid hemorrhage from right posterior communicating artery
			I60.32	Nontraumatic subarachnoid hemorrhage from left posterior communicating artery
			I60.4	Nontraumatic subarachnoid hemorrhage from basilar artery
			I60.50	Nontraumatic subarachnoid hemorrhage from unspecified vertebral artery
			I60.51	Nontraumatic subarachnoid hemorrhage from right vertebral artery
			I60.52	Nontraumatic subarachnoid hemorrhage from left vertebral artery
			I60.6	Nontraumatic subarachnoid hemorrhage from other intracranial arteries
			I60.7	Nontraumatic subarachnoid hemorrhage from unspecified intracranial artery
			I60.8	Other nontraumatic subarachnoid hemorrhage
			I60.9	Nontraumatic subarachnoid hemorrhage, unspecified
			I61.0	Nontraumatic intracerebral hemorrhage in hemisphere, subcortical
			I61.1	Nontraumatic intracerebral hemorrhage in hemisphere, cortical
			I61.2	Nontraumatic intracerebral hemorrhage in hemisphere, unspecified
			I61.3	Nontraumatic intracerebral hemorrhage in brain stem
			I61.4	Nontraumatic intracerebral hemorrhage in cerebellum
			I61.5	Nontraumatic intracerebral hemorrhage, intraventricular
			I61.6	Nontraumatic intracerebral hemorrhage, multiple localized
			I61.8	Other nontraumatic intracerebral hemorrhage
			I61.9	Nontraumatic intracerebral hemorrhage, unspecified
			I62.00	Nontraumatic subdural hemorrhage, unspecified
			I62.01	Nontraumatic acute subdural hemorrhage

© 2023 Fink DA et al. *JAMA Network Open.*

ICD-9-CM	Descriptions	ICD-10-CM	Descriptions
		I62.02	Nontraumatic subacute subdural hemorrhage
		I62.03	Nontraumatic chronic subdural hemorrhage
		I62.1	Nontraumatic extradural hemorrhage
		I62.9	Nontraumatic intracranial hemorrhage, unspecified
		I63.00	Cerebral infarction due to thrombosis of unspecified precerebral artery
		I63.011	Cerebral infarction due to thrombosis of right vertebral artery
		I63.012	Cerebral infarction due to thrombosis of left vertebral artery
		I63.013	Cerebral infarction due to thrombosis of bilateral vertebral arteries
		I63.019	Cerebral infarction due to thrombosis of unspecified vertebral artery
		I63.02	Cerebral infarction due to thrombosis of basilar artery
		I63.031	Cerebral infarction due to thrombosis of right carotid artery
		I63.032	Cerebral infarction due to thrombosis of left carotid artery
		I63.033	Cerebral infarction due to thrombosis of bilateral carotid arteries
		I63.039	Cerebral infarction due to thrombosis of unspecified carotid artery
		I63.09	Cerebral infarction due to thrombosis of other precerebral artery
		I63.10	Cerebral infarction due to embolism of unspecified precerebral artery
		I63.111	Cerebral infarction due to embolism of right vertebral artery
		I63.112	Cerebral infarction due to embolism of left vertebral artery
		I63.113	Cerebral infarction due to embolism of bilateral vertebral arteries
		I63.119	Cerebral infarction due to embolism of unspecified vertebral artery
		I63.12	Cerebral infarction due to embolism of basilar artery
		I63.131	Cerebral infarction due to embolism of right carotid artery
		I63.132	Cerebral infarction due to embolism of left carotid artery
		I63.133	Cerebral infarction due to embolism of bilateral carotid arteries
		I63.139	Cerebral infarction due to embolism of unspecified carotid artery
		I63.19	Cerebral infarction due to embolism of other precerebral artery
		I63.20	Cerebral infarction due to unspecified occlusion or stenosis of unspecified precerebral arteries
		I63.211	Cerebral infarction due to unspecified occlusion or stenosis of right vertebral arteries
		I63.212	Cerebral infarction due to unspecified occlusion or stenosis of left vertebral arteries

© 2023 Fink DA et al. *JAMA Network Open.*

ICD-9-CM	Descriptions	ICD-10-CM	Descriptions
		I63.213	Cerebral infarction due to unspecified occlusion or stenosis of bilateral vertebral arteries
		I63.219	Cerebral infarction due to unspecified occlusion or stenosis of unspecified vertebral arteries
		I63.22	Cerebral infarction due to unspecified occlusion or stenosis of basilar arteries
		I63.231	Cerebral infarction due to unspecified occlusion or stenosis of right carotid arteries
		I63.232	Cerebral infarction due to unspecified occlusion or stenosis of left carotid arteries
		I63.233	Cerebral infarction due to unspecified occlusion or stenosis of bilateral carotid arteries
		I63.239	Cerebral infarction due to unspecified occlusion or stenosis of unspecified carotid arteries
		I63.29	Cerebral infarction due to unspecified occlusion or stenosis of other precerebral arteries
		I63.30	Cerebral infarction due to thrombosis of unspecified cerebral artery
		I63.311	Cerebral infarction due to thrombosis of right middle cerebral artery
		I63.312	Cerebral infarction due to thrombosis of left middle cerebral artery
		I63.313	Cerebral infarction due to thrombosis of bilateral middle cerebral arteries
		I63.319	Cerebral infarction due to thrombosis of unspecified middle cerebral artery
		I63.321	Cerebral infarction due to thrombosis of right anterior cerebral artery
		I63.322	Cerebral infarction due to thrombosis of left anterior cerebral artery
		I63.323	Cerebral infarction due to thrombosis of bilateral anterior arteries
		I63.329	Cerebral infarction due to thrombosis of unspecified anterior cerebral artery
		I63.331	Cerebral infarction due to thrombosis of right posterior cerebral artery

© 2023 Fink DA et al. *JAMA Network Open.*

ICD-9-CM	Descriptions	ICD-10-CM	Descriptions
		I63.332	Cerebral infarction due to thrombosis of left posterior cerebral artery
		I63.333	Cerebral infarction to thrombosis of bilateral posterior arteries
		I63.339	Cerebral infarction due to thrombosis of unspecified posterior cerebral artery
		I63.341	Cerebral infarction due to thrombosis of right cerebellar artery
		I63.342	Cerebral infarction due to thrombosis of left cerebellar artery
		I63.343	Cerebral infarction to thrombosis of bilateral cerebellar arteries
		I63.349	Cerebral infarction due to thrombosis of unspecified cerebellar artery
		I63.39	Cerebral infarction due to thrombosis of other cerebral artery
		I63.40	Cerebral infarction due to embolism of unspecified cerebral artery
		I63.411	Cerebral infarction due to embolism of right middle cerebral artery
		I63.412	Cerebral infarction due to embolism of left middle cerebral artery
		I63.413	Cerebral infarction due to embolism of bilateral middle cerebral arteries
		I63.419	Cerebral infarction due to embolism of unspecified middle cerebral artery
		I63.421	Cerebral infarction due to embolism of right anterior cerebral artery
		I63.422	Cerebral infarction due to embolism of left anterior cerebral artery
		I63.423	Cerebral infarction due to embolism of bilateral anterior cerebral arteries
		I63.429	Cerebral infarction due to embolism of unspecified anterior cerebral artery
		I63.431	Cerebral infarction due to embolism of right posterior cerebral artery
		I63.432	Cerebral infarction due to embolism of left posterior cerebral artery
		I63.433	Cerebral infarction due to embolism of bilateral posterior cerebral arteries
		I63.439	Cerebral infarction due to embolism of unspecified posterior cerebral artery
		I63.441	Cerebral infarction due to embolism of right cerebellar artery
		I63.442	Cerebral infarction due to embolism of left cerebellar artery
		I63.443	Cerebral infarction due to embolism of bilateral cerebellar arteries

© 2023 Fink DA et al. *JAMA Network Open.*

ICD-9-CM	Descriptions	ICD-10-CM	Descriptions
		I63.449	Cerebral infarction due to embolism of unspecified cerebellar artery
		I63.49	Cerebral infarction due to embolism of other cerebral artery
		I63.50	Cerebral infarction due to unspecified occlusion or stenosis of unspecified cerebral artery
		I63.511	Cerebral infarction due to unspecified occlusion or stenosis of right middle cerebral artery
		I63.512	Cerebral infarction due to unspecified occlusion or stenosis of left middle cerebral artery
		I63.513	Cerebral infarction due to unspecified occlusion or stenosis of bilateral middle arteries
		I63.519	Cerebral infarction due to unspecified occlusion or stenosis of unspecified middle cerebral art
		I63.521	Cerebral infarction due to unspecified occlusion or stenosis of right anterior cerebral artery
		I63.522	Cerebral infarction due to unspecified occlusion or stenosis of left anterior cerebral artery
		I63.523	Cerebral infarction due to unspecified occlusion or stenosis of bilateral anterior arteries
		I63.529	Cerebral infarction due to unspecified occlusion or stenosis of unspecified anterior cerebral a
		I63.531	Cerebral infarction due to unspecified occlusion or stenosis of right posterior cerebral artery
		I63.532	Cerebral infarction due to unspecified occlusion or stenosis of left posterior cerebral artery
		I63.533	Cerebral infarction due to unspecified occlusion or stenosis of bilateral posterior arteries
		I63.539	Cerebral infarction due to unspecified occlusion or stenosis of unspecified posterior cerebral
		I63.541	Cerebral infarction due to unspecified occlusion or stenosis of right cerebellar artery
		I63.542	Cerebral infarction due to unspecified occlusion or stenosis of left cerebellar artery
		I63.543	Cerebral infarction due to unspecified occlusion or stenosis of bilateral cerebellar arteries

© 2023 Fink DA et al. *JAMA Network Open.*

ICD-9-CM	Descriptions	ICD-10-CM	Descriptions
		I63.549	Cerebral infarction due to unspecified occlusion or stenosis of unspecified cerebellar artery
		I63.59	Cerebral infarction due to unspecified occlusion or stenosis of other cerebral artery
		I63.6	Cerebral infarction due to cerebral venous thrombosis, nonpyogenic
		I63.8	Other cerebral infarction
		I63.81	Other cerebral infarction due to occlusion or stenosis of small artery
		I63.89	Other cerebral infarction
		I63.9	Cerebral infarction, unspecified
		I65.01	Occlusion and stenosis of right vertebral artery
		I65.02	Occlusion and stenosis of left vertebral artery
		I65.03	Occlusion and stenosis of bilateral vertebral arteries
		I65.09	Occlusion and stenosis of unspecified vertebral artery
		I65.1	Occlusion and stenosis of basilar artery
		I65.21	Occlusion and stenosis of right carotid artery
		I65.22	Occlusion and stenosis of left carotid artery
		I65.23	Occlusion and stenosis of bilateral carotid arteries
		I65.29	Occlusion and stenosis of unspecified carotid artery
		I65.8	Occlusion and stenosis of other precerebral arteries
		I65.9	Occlusion and stenosis of unspecified precerebral artery
		I66.01	Occlusion and stenosis of right middle cerebral artery
		I66.02	Occlusion and stenosis of left middle cerebral artery
		I66.03	Occlusion and stenosis of bilateral middle cerebral arteries
		I66.09	Occlusion and stenosis of unspecified middle cerebral artery
		I66.11	Occlusion and stenosis of right anterior cerebral artery
		I66.12	Occlusion and stenosis of left anterior cerebral artery
		I66.13	Occlusion and stenosis of bilateral anterior cerebral arteries
		I66.19	Occlusion and stenosis of unspecified anterior cerebral artery
		I66.21	Occlusion and stenosis of right posterior cerebral artery
		I66.22	Occlusion and stenosis of left posterior cerebral artery
		I66.23	Occlusion and stenosis of bilateral posterior cerebral arteries
		I66.29	Occlusion and stenosis of unspecified posterior cerebral artery
		I66.3	Occlusion and stenosis of cerebellar arteries

© 2023 Fink DA et al. *JAMA Network Open.*

ICD-9-CM	Descriptions	ICD-10-CM	Descriptions
		I66.8	Occlusion and stenosis of other cerebral arteries
		I66.9	Occlusion and stenosis of unspecified cerebral artery
		I67.0	Dissection of cerebral arteries, nonruptured
		I67.1	Cerebral aneurysm, nonruptured
		I67.2	Cerebral atherosclerosis
		I67.3	Progressive vascular leukoencephalopathy
		I67.4	Hypertensive encephalopathy
		I67.5	Moyamoya disease
		I67.6	Nonpyogenic thrombosis of intracranial venous system
		I67.7	Cerebral arteritis, not elsewhere classified
		I67.81	Acute cerebrovascular insufficiency
		I67.82	Cerebral ischemia
		I67.83	Posterior reversible encephalopathy syndrome
		I67.841	Reversible cerebrovascular vasoconstriction syndrome
		I67.848	Other cerebrovascular vasospasm and vasoconstriction
		I67.89	Other cerebrovascular disease
		I67.9	Cerebrovascular disease, unspecified
		I68.0	Cerebral amyloid angiopathy
		I68.2	Cerebral arteritis in other diseases classified elsewhere
		I68.8	Other cerebrovascular disorders in diseases classified elsewhere
		O22.52	Cerebral venous thrombosis in pregnancy second trimester
		O22.53	Cerebral venous thrombosis in pregnancy third trimester
		O87.3	Cerebral venous thrombosis in the puerperium
		I97.810	Intraoperative cerebrovascular infarction during cardiac surgery
		I97.811	Intraoperative cerebrovascular infarction during other surgery
		I97.820	Postprocedural cerebrovascular infarction during cardiac surgery
		I97.821	Postprocedural cerebrovascular infarction during other surgery
**Acute Heart Failure / Pulmonary edema Diagnosis**			
428.0	Congestive heart failure, unspecified	J81.0	Acute pulmonary edema
428.1	Left heart failure	I50.1	Left ventricular failure
428.21	Acute systolic heart failure	I50.20	Unspecified systolic (congestive) heart failure

© 2023 Fink DA et al. *JAMA Network Open.*

	ICD-9-CM	Descriptions	ICD-10-CM	Descriptions
	428.23	Acute on chronic systolic heart failure	I50.21	Acute systolic (congestive) heart failure
	428.31	Acute diastolic heart failure	I50.23	Acute on chronic systolic (congestive) heart failure
	428.33	Acute on chronic diastolic heart failure	I50.30	Unspecified diastolic (congestive) heart failure
	428.41	Acute combined systolic and diastolic heart failure	I50.31	Acute diastolic (congestive) heart failure
	428.43	Acute on chronic combined systolic and diastolic heart failure	I50.33	Acute on chronic diastolic (congestive) heart failure
	518.4	Acute edema of lung, unspecified	I50.40	Unspecified combined systolic (congestive) and diastolic (congestive) heart failure
			I50.41	Acute combined systolic (congestive) and diastolic (congestive) heart failure
			I50.43	Acute on chronic combined systolic (congestive) and diastolic (congestive) heart failure
			I50.9	Heart failure, unspecified
**Severe anesthesia complications Diagnosis**				
	668.0x	Pulmonary complications of anesthesia or other sedation in labor and delivery	O74.0	Aspiration pneumonitis due to anesthesia during labor and delivery
	668.1x	Cardiac complications of anesthesia or	O74.1	Other pulmonary complications of anesthesia during labor and delivery

© 2023 Fink DA et al. *JAMA Network Open.*

	ICD-9-CM	Descriptions	ICD-10-CM	Descriptions
		other sedation in labor and delivery Central nervous system complications of anesthesia or other sedation in labor and delivery		
	668.2x		O74.2	Cardiac complications of anesthesia during labor and delivery
			O74.3	Central nervous system complications of anesthesia during labor and delivery
			O89.01	Aspiration pneumonitis due to anesthesia during the puerperium
			O89.09	Other pulmonary complications of anesthesia during the puerperium
			O89.1	Cardiac complications of anesthesia during the puerperium
			O89.2	Central nervous system complications of anesthesia during the puerperium
**Sepsis Diagnosis**				
	038.xx	Septicemia	O85	Puerperal sepsis
	995.91	Sepsis	O86.04	Sepsis following an obstetrical procedure
	995.92	Severe sepsis	T80.211A	Bloodstream infection due to central venous catheter, initial encounter
			T81.4XXA	Infection following a procedure, initial encounter
	670.22	Puerperal sepsis, delivered, with mention of postpartum complication	T81.44XA	Sepsis following a procedure, initial encounter
	670.24	Puerperal sepsis, postpartum condition or complication	T81.44XD	Sepsis following a procedure, subsequent encounter

© 2023 Fink DA et al. *JAMA Network Open.*

	ICD-9-CM	Descriptions	ICD-10-CM	Descriptions
	998.59	Other postoperative infection	T81.44XS	Sepsis following a procedure, sequela
	999.32	Bloodstream infection due to central venous catheter	T81.44	Sepsis following a procedure
	998.02	Postoperative shock, septic	R65.20	Severe sepsis without septic shock
	785.52	Septic shock	A40.0	Sepsis due to streptococcus, group A
	449	Septic arterial embolism	A40.1	Sepsis due to streptococcus, group B
			A40.3	Sepsis due to Streptococcus pneumoniae
			A40.8	Other streptococcal sepsis
			A40.9	Streptococcal sepsis, unspecified
			A41.01	Sepsis due to Methicillin susceptible Staphylococcus aureus
			A41.02	Sepsis due to Methicillin resistant Staphylococcus aureus
			A41.1	Sepsis due to other specified staphylococcus
			A41.2	Sepsis due to unspecified staphylococcus
			A41.3	Sepsis due to Hemophilus influenza
			A41.4	Sepsis due to anaerobes
			A41.50	Gram-negative sepsis, unspecified
			A41.51	Sepsis due to Escherichia coli
			A41.52	Sepsis due to pseudomonas
			A41.53	Sepsis due to serratia
			A41.59	Other gram-negative sepsis
			A41.81	Sepsis due to Enterococcus
			A41.89	Other specified sepsis
			A41.9	Sepsis, unspecified
			A32.7	Listerial sepsis
**Shock Diagnosis**				
	669.1x	Obstetric shock	O75.1	Shock during or following labor and delivery
	785.5x	Shock without mention of trauma	R57.0	Cardiogenic shock

© 2023 Fink DA et al. *JAMA Network Open.*

ICD-9-CM	Descriptions	ICD-10-CM	Descriptions
995.0	Other anaphylactic reaction	R57.1	Hypovolemic shock
995.4	Shock due to anesthesia, not elsewhere classified	R57.8	Other shock
998.0x	Postoperative shock not elsewhere classified	R57.9	Shock, unspecified
		R65.21	Severe sepsis with septic shock
		T78.2XXA	Anaphylactic shock, unspecified
		T88.2XXA	Shock due to anesthesia
		T88.6XXA	Anaphylactic reaction due to adverse effect of correct drug or medicament properly administered
		T81.10XA	Postprocedural shock unspecified, initial encounter
		T81.11XA	Postprocedural cardiogenic shock, initial encounter
		T81.19XA	Other postprocedural shock, initial encounter
**Sickle Cell Disease with Crisis Diagnosis**			
282.42	Sickle-cell thalassemia with crisis	D57.00	Hb-SS disease with crisis, unspecified
282.62	Hb-SS disease with crisis	D57.01	Hb-SS disease with acute chest syndrome
282.64		D57.02	Hb-SS disease with splenic sequestration
282.69	Other sickle-cell disease with crisis	D57.03	Hb-SS disease with cerebral vascular involvement
		D57.09	Hb-SS disease with crisis with other specified complication
		D57.211	Sickle-cell/Hb-C disease with acute chest syndrome
		D57.212	Sickle-cell/Hb-C disease with splenic sequestration
		D57.219	Sickle-cell/Hb-C disease with crisis, unspecified
		D57.213	Sickle-cell/Hb-C disease with cerebral vascular involvement
		D57.218	Sickle-cell/Hb-C disease with crisis with other specified complica
		D57.411	Sickle-cell thalassemia with acute chest syndrome

© 2023 Fink DA et al. *JAMA Network Open.*

ICD-9-CM	Descriptions	ICD-10-CM	Descriptions
		D57.412	Sickle-cell thalassemia with splenic sequestration
		D57.419	Sickle-cell thalassemia with crisis, unspecified
		D57.413	Sickle-cell thalassemia, unspecified, with cerebral vascular invol
		D57.418	Sickle-cell thalassemia, unspecified, with crisis with other speci
		D57.811	Other sickle-cell disorders with acute chest syndrome
		D57.812	Other sickle-cell disorders with splenic sequestration
		D57.813	Other sickle-cell disorders with cerebral vascular involvement
		D57.818	Other sickle-cell disorders with crisis with other specified compl
		D57.819	Other sickle-cell disorders with crisis, unspecified
**Air and thrombotic embolism Diagnosis**	Sickle-cell/Hb-C disease with crisis		
415.1x	Pulmonary embolism and infarction	I26.01	Septic pulmonary embolism with acute cor pulmonale
673.0x	Obstetrical air embolism	I26.02	Saddle embolus of pulmonary artery with acute cor pulmonale
673.2x	Obstetrical blood-clot embolism	I26.09	Other pulmonary embolism with acute cor pulmonale
673.3x	Obstetrical pyemic and septic embolism	I26.90	Septic pulmonary embolism without acute cor pulmonale
673.8x	Other obstetrical pulmonary embolism	I26.92	Saddle embolus of pulmonary artery without acute cor pulmonale
		I26.99	Other pulmonary embolism without acute cor pulmonale
		O88.012	Air embolism in pregnancy, second trimester
		O88.013	Air embolism in pregnancy, third trimester
		O88.019	Air embolism in pregnancy, unspecified trimester
		O88.02	Air embolism in childbirth
		O88.03	Air embolism in the puerperium
		O88.212	Thromboembolism in pregnancy, second trimester
		O88.213	Thromboembolism in pregnancy, third trimester
		O88.219	Thromboembolism in pregnancy, unspecified trimester

© 2023 Fink DA et al. *JAMA Network Open.*

ICD-9-CM	Descriptions	ICD-10-CM	Descriptions
		O88.22	Thromboembolism in childbirth
		O88.23	Thromboembolism in the puerperium
		O88.312	Pyemic and septic embolism in pregnancy, second trimester
		O88.313	Pyemic and septic embolism in pregnancy, third trimester
		O88.319	Pyemic and septic embolism in pregnancy, unspecified trimester
		O88.32	Pyemic and septic embolism in childbirth
		O88.33	Pyemic and septic embolism in the puerperium
		O88.812	Other embolism in pregnancy second trimester
		O88.813	Other embolism in pregnancy third trimester
		O88.819	Other embolism in pregnancy, unspecified trimester
		O88.82	Other embolism in childbirth
		O88.83	Other embolism in the puerperium
**Conversion of cardiac rhythm Procedure**			
99.6x	Conversion Of Cardiac Rhythm	5A2204Z	Restoration of Cardiac Rhythm, Single Cardiac countershock with successful conversion to sinus rhythm ICD-9: 99.61,2,4,9
		5A12012	Performance of Cardiac Output, Single, Manual
**Hysterectomy Procedure**			
68.3x	Subtotal Abdominal Hysterectomy	0UT90ZZ	Resection of Uterus, Open Approach
68.4x	Total Abdominal Hysterectomy		
68.5x	Vaginal Hysterectomy	0UT97ZZ	Resection of Uterus, Via Natural or Artificial Opening
68.6x	Radical Abdominal Hysterectomy		
68.7x	Radical Vaginal Hysterectomy		
68.8x	Pelvic evisceration	0UT90ZL	Resection of Uterus, Supracervical, Open Approach
68.9x	Other and unspecified hysterectomy	0UT97ZL	Resection of Uterus, Supracervical, Via Natural or Artificial Opening

ICD-9-CM	Descriptions	ICD-10-CM	Descriptions
**Temporary tracheostomy Procedure**			
31.1	Temporary tracheostomy		
		0B110F4	Bypass Trachea to Cutaneous with Tracheostomy Device, Open Approach
		0B113F4	Bypass Trachea to Cutaneous with Tracheostomy Device, Percutaneous Approach
		0B114F4	Bypass Trachea to Cutaneous with Tracheostomy Device, Percutaneous Endoscopic Approach
**Ventilation Procedure**			
93.90	Non-invasive mechanical ventilation	5A1935Z	Respiratory Ventilation, Less than 24 Consecutive Hours
96.01	Insertion of nasopharyngeal airway	5A1945Z	Respiratory Ventilation, 24-96 Consecutive Hours
96.02	Insertion of oropharyngeal airway	5A1955Z	Respiratory Ventilation, Greater than 96 Consecutive Hours
96.03	Insertion of esophageal obturator airway		
96.05	Other intubation of respiratory tract		
**Blood transfusion Procedure**			
99.0x	Transfusion Of Blood And Blood Components	30230H0	Transfusion of Autologous Whole Blood into Peripheral Vein, Open Approach
		30230K0	Transfusion of Autologous Frozen Plasma into Peripheral Vein, Open Approach
		30230L0	Transfusion of Autologous Fresh Plasma into Peripheral Vein, Open Approach
		30230M0	Transfusion of Autologous Plasma Cryoprecipitate into Peripheral Vein, Open Approach

© 2023 Fink DA et al. *JAMA Network Open.*

ICD-9-CM	Descriptions	ICD-10-CM	Descriptions
		30230N0	Transfusion of Autologous Red Blood Cells into Peripheral Vein, Open Approach
		30230P0	Transfusion of Autologous Frozen Red Cells into Peripheral Vein, Open Approach
		30230R0	Transfusion of Autologous Platelets into Peripheral Vein, Open Approach
		30230T0	Transfusion of Autologous Fibrinogen into Peripheral Vein, Open Approach
		30230H1	Transfusion of Non-autologous Whole Blood into Peripheral Vein, Open Approach
		30230K1	Transfusion of Non-autologous Frozen Plasma into Peripheral Vein, Open Approach
		30230L1	Transfusion of Non-autologous Fresh Plasma into Peripheral Vein, Open Approach
		30230M1	Transfusion of Non-autologous Plasma Cryoprecipitate into Peripheral Vein, Open Approach
		30230N1	Transfusion of Non-autologous Red Blood Cells into Peripheral Vein, Open Approach
		30230P1	Transfusion of Non-autologous Frozen Red Cells into Peripheral Vein, Open Approach
		30230R1	Transfusion of Non-autologous Platelets into Peripheral Vein, Open Approach
		30230T1	Transfusion of Non-autologous Fibrinogen into Peripheral Vein, Open Approach
		30233H0	Transfusion of Autologous Whole Blood into Peripheral Vein, Percutaneous Approach
		30233K0	Transfusion of Autologous Frozen Plasma into Peripheral Vein, Percutaneous Approach
		30233L0	Transfusion of Autologous Fresh Plasma into Peripheral Vein, Percutaneous Approach
		30233M0	Transfusion of Autologous Plasma Cryoprecipitate into Peripheral Vein, Percutaneous Approach
		30233N0	Transfusion of Autologous Red Blood Cells into Peripheral Vein, Percutaneous Approach

© 2023 Fink DA et al. *JAMA Network Open.*

ICD-9-CM	Descriptions	ICD-10-CM	Descriptions
		30233P0	Transfusion of Autologous Frozen Red Cells into Peripheral Vein, Percutaneous Approach
		30233R0	Transfusion of Autologous Platelets into Peripheral Vein, Percutaneous Approach
		30233T0	Transfusion of Autologous Fibrinogen into Peripheral Vein, Percutaneous Approach
		30233H1	Transfusion of Non-autologous Whole Blood into Peripheral Vein, Percutaneous Approach
		30233K1	Transfusion of Non-autologous Frozen Plasma into Peripheral Vein, Percutaneous Approach
		30233L1	Transfusion of Non-autologous Fresh Plasma into Peripheral Vein, Percutaneous Approach
		30233M1	Transfusion of Non-autologous Plasma Cryoprecipitate into Peripheral Vein, Percutaneous Approach
		30233N1	Transfusion of Non-autologous Red Blood Cells into Peripheral Vein, Percutaneous Approach
		30233P1	Transfusion of Non-autologous Frozen Red Cells into Peripheral Vein, Percutaneous Approach
		30233R1	Transfusion of Non-autologous Platelets into Peripheral Vein, Percutaneous Approach
		30233T1	Transfusion of Non-autologous Fibrinogen into Peripheral Vein, Percutaneous Approach
		30240H0	Transfusion of Autologous Whole Blood into Central Vein, Open Approach
		30240K0	Transfusion of Autologous Frozen Plasma into Central Vein, Open Approach
		30240L0	Transfusion of Autologous Fresh Plasma into Central Vein, Open Approach
		30240M0	Transfusion of Autologous Plasma Cryoprecipitate into Central Vein, Open Approach
		30240N0	Transfusion of Autologous Red Blood Cells into CentralVein, Open Approach
		30240P0	Transfusion of Autologous Frozen Red Cells into Central Vein, Open Approach

© 2023 Fink DA et al. *JAMA Network Open.*

ICD-9-CM	Descriptions	ICD-10-CM	Descriptions
		30240R0	Transfusion of Autologous Platelets into Central Vein, Open Approach
		30240T0	Transfusion of Autologous Fibrinogen into Central Vein, Open Approach
		30240H1	Transfusion of Non-autologous Whole Blood into Central Vein, Open Approach
		30240K1	Transfusion of Non-autologous Frozen Plasma into Central Vein, Open Approach
		30240L1	Transfusion of Non-autologous Fresh Plasma into Central Vein, Open Approach
		30240M1	Transfusion of Non-autologous Plasma Cryoprecipitate into Central Vein, Open Approach
		30240N1	Transfusion of Non-autologous Red Blood Cells into Central Vein, Open Approach
		30240P1	Transfusion of Non-autologous Frozen Red Cells into Central Vein, Open Approach
		30240R1	Transfusion of Non-autologous Platelets into Central Vein, Open Approach
		30240T1	Transfusion of Non-autologous Fibrinogen into Central Vein, Open Approach
		30243H0	Transfusion of Autologous Whole Blood into Central Vein, Percutaneous Approach
		30243K0	Transfusion of Autologous Frozen Plasma into Central Vein, Percutaneous Approach
		30243L0	Transfusion of Autologous Fresh Plasma into Central Vein, Percutaneous Approach
		30243M0	Transfusion of Autologous Plasma Cryoprecipitate into Central Vein, Percutaneous Approach
		30243N0	Transfusion of Autologous Red Blood Cells into Central Vein, Percutaneous Approach
		30243P0	Transfusion of Autologous Frozen Red Cells into Central Vein, Percutaneous Approach
		30243R0	Transfusion of Autologous Platelets into Centrall Vein, Percutaneous Approach

© 2023 Fink DA et al. *JAMA Network Open.*

ICD-9-CM	Descriptions	ICD-10-CM	Descriptions
		30243T0	Transfusion of Autologous Fibrinogen into Central Vein, Percutaneous Approach
		30243H1	Transfusion of Non-autologous Whole Blood into Central Vein, Percutaneous Approach
		30243K1	Transfusion of Non-autologous Frozen Plasma into Central Vein, Percutaneous Approach
		30243L1	Transfusion of Non-autologous Fresh Plasma into Central Vein, Percutaneous Approach
		30243M1	Transfusion of Non-autologous Plasma Cryoprecipitate into Central Vein, Percutaneous Approach
		30243N1	Transfusion of Non-autologous Red Blood Cells into Central Vein, Percutaneous Approach
		30243P1	Transfusion of Non-autologous Frozen Red Cells into Central Vein, Percutaneous Approach
		30243R1	Transfusion of Non-autologous Platelets into Central Vein, Percutaneous Approach
		30243T1	Transfusion of Non-autologous Fibrinogen into Central Vein, Percutaneous Approach
		30250H0	Transfusion of Autologous Whole Blood into Peripheral Artery, Open Approach
		30250K0	Transfusion of Autologous Frozen Plasma into Peripheral Artery, Open Approach
		30250L0	Transfusion of Autologous Fresh Plasma into Peripheral Artery, Open Approach
		30250M0	Transfusion of Autologous Plasma Cryoprecipitate into Artery Vein, Open Approach
		30250N0	Transfusion of Autologous Red Blood Cells into Artery Vein, Open Approach
		30250P0	Transfusion of Autologous Frozen Red Cells into Artery Vein, Open Approach
		30250R0	Transfusion of Autologous Platelets into Artery Vein, Open Approach
		30250T0	Transfusion of Autologous Fibrinogen into Artery Vein, Open Approach

© 2023 Fink DA et al. *JAMA Network Open.*

ICD-9-CM	Descriptions	ICD-10-CM	Descriptions
		30250H1	Transfusion of Non-autologous Whole Blood into Peripheral Artery, Open Approach
		30250K1	Transfusion of Non-autologous Frozen Plasma into Peripheral Artery, Open Approach
		30250L1	Transfusion of Non-autologous Fresh Plasma into PeripheralArtery, Open Approach
		30250M1	Transfusion of Non-autologous Plasma Cryoprecipitate into Peripheral Artery, Open Approach
		30250N1	Transfusion of Non-autologous Red Blood Cells into Peripheral Artery, Open Approach
		30250P1	Transfusion of Non-autologous Frozen Red Cells into Peripheral Artery, Open Approach
		30250R1	Transfusion of Non-autologous Platelets into Peripheral Artery, Open Approach
		30250T1	Transfusion of Non-autologous Fibrinogen into Peripheral Artery, Open Approach
		30253H0	Transfusion of Autologous Whole Blood into Peripheral Vein, Percutaneous Approach
		30253K0	Transfusion of Autologous Frozen Plasma into Peripheral Artery, Percutaneous Approach
		30253L0	Transfusion of Autologous Fresh Plasma into Peripheral Artery, Percutaneous Approach
		30253M0	Transfusion of Autologous Plasma Cryoprecipitate into Peripheral Artery, Percutaneous Approach
		30253N0	Transfusion of Autologous Red Blood Cells into PeripheralArtery, Percutaneous Approach
		30253P0	Transfusion of Autologous Frozen Red Cells into Peripheral Artery, Percutaneous Approach
		30253R0	Transfusion of Autologous Platelets into Peripheral Artery, Percutaneous Approach
		30253T0	Transfusion of Autologous Fibrinogen into Peripheral Artery, Percutaneous Approach
		30253H1	Transfusion of Non-autologous Whole Blood into Peripheral Artery, Percutaneous Approach

© 2023 Fink DA et al. *JAMA Network Open.*

ICD-9-CM	Descriptions	ICD-10-CM	Descriptions
		30253K1	Transfusion of Non-autologous Frozen Plasma into Peripheral Artery, Percutaneous Approach
		30253L1	Transfusion of Non-autologous Fresh Plasma into Peripheral Artery, Percutaneous Approach
		30253M1	Transfusion of Non-autologous Plasma Cryoprecipitate into Peripheral Artery, Percutaneous Approach
		30253N1	Transfusion of Non-autologous Red Blood Cells into Peripheral Artery, Percutaneous Approach
		30253P1	Transfusion of Non-autologous Frozen Red Cells into Peripheral Artery, Percutaneous Approach
		30253R1	Transfusion of Non-autologous Platelets into Peripheral Artery, Percutaneous Approach
		30253T1	Transfusion of Non-autologous Fibrinogen into Peripheral Artery, Percutaneous Approach
		30260H0	Transfusion of Autologous Whole Blood into Central Artery, Open Approach
		30260K0	Transfusion of Autologous Frozen Plasma into Central Artery, Open Approach
		30260L0	Transfusion of Autologous Fresh Plasma into Central Artery, Open Approach
		30260M0	Transfusion of Autologous Plasma Cryoprecipitate into Central Artery, Open Approach
		30260N0	Transfusion of Autologous Red Blood Cells into Central Artery, Open Approach
		30260P0	Transfusion of Autologous Frozen Red Cells into Central Artery, Open Approach
		30260R0	Transfusion of Autologous Platelets into Central Artery, Open Approach
		30260T0	Transfusion of Autologous Fibrinogen into Central Artery, Open Approach
		30260H1	Transfusion of Non-autologous Whole Blood into Central Artery, Open Approach
		30260K1	Transfusion of Non-autologous Frozen Plasma into Central Artery, Open Approach

© 2023 Fink DA et al. *JAMA Network Open.*

ICD-9-CM	Descriptions	ICD-10-CM	Descriptions
		30260L1	Transfusion of Non-autologous Fresh Plasma into Central Artery, Open Approach
		30260M1	Transfusion of Non-autologous Plasma Cryoprecipitate into Central Artery, Open Approach
		30260N1	Transfusion of Non-autologous Red Blood Cells into Central Artery, Open Approach
		30260P1	Transfusion of Non-autologous Frozen Red Cells into Central Artery, Open Approach
		30260R1	Transfusion of Non-autologous Platelets into Central Artery, Open Approach
		30260T1	Transfusion of Non-autologous Fibrinogen into Central Artery, Open Approach
		30263H0	Transfusion of Autologous Whole Blood into Central Artery, Percutaneous Approach
		30263K0	Transfusion of Autologous Frozen Plasma into Central Artery, Percutaneous Approach
		30263L0	Transfusion of Autologous Fresh Plasma into Central Artery, Percutaneous Approach
		30263M0	Transfusion of Autologous Plasma Cryoprecipitate into Central Artery, Percutaneous Approach
		30263N0	Transfusion of Autologous Red Blood Cells into Central Artery, Percutaneous Approach
		30263P0	Transfusion of Autologous Frozen Red Cells into Central Artery, Percutaneous Approach
		30263R0	Transfusion of Autologous Platelets into Centrall Artery, Percutaneous Approach
		30263T0	Transfusion of Autologous Fibrinogen into Central Artery, Percutaneous Approach
		30263H1	Transfusion of Non-autologous Whole Blood into Central Artery, Percutaneous Approach
		30263K1	Transfusion of Non-autologous Frozen Plasma into Central Artery, Percutaneous Approach
		30263L1	Transfusion of Non-autologous Fresh Plasma into Central Artery, Percutaneous Approach

© 2023 Fink DA et al. *JAMA Network Open.*

ICD-9-CM	Descriptions	ICD-10-CM	Descriptions
		30263M1	Transfusion of Non-autologous Plasma Cryoprecipitate into Central Artery, Percutaneous Approach
		30263N1	Transfusion of Non-autologous Red Blood Cells into Central Artery, Percutaneous Approach
		30263P1	Transfusion of Non-autologous Frozen Red Cells into Central Artery Percutaneous Approach
		30263R1	Transfusion of Non-autologous Platelets into Central Artery, Percutaneous Approach
		30263T1	Transfusion of Non-autologous Fibrinogen into Central Artery, Percutaneous Approach

eTable 3. *ICD-9-CM* and *ICD-10-CM* Diagnosis Codes Used to Identify Maternal Comorbidities

Comorbid Conditions	ICD-9-CM	ICD-10-CM
Pulmonary hypertension	416.0x, 416.8x, 416.9x	I27.0,I27.2x,I27.81,I27.9
Placenta previa	641.0x, 641.1x	O44.x
Sickle cell disease	282.4x, 282.6x	D57.x
Gestational hypertension	642.3x (without preeclampsia/eclampsia or pre-existing hypertension)	O13.x
Mild preeclampsia or unspecified preeclampsia	642.4x, 642.7x (without severe preeclampsia/eclampsia)	O14.0x,O14.9x
Severe preeclampsia	642.5x,642.6xx	O14.1x
Chronic renal disease	581.x–583.x, 585.x, 587.x, 588.x, 646.2x	N04.x,N03.x,N05.x,N18.x,N26.9,N25.x,O26.83
Preexisting hypertension	642.0x–642.2x, 642.7x	I10.x,I11.x,I12.x,I13.x,I15.x,O10.x
Chronic ischemic heart disease	412.x–414.x	I25.x, I20.x
Congenital heart disease	745.0x–747.4x, 648.5x	Q20.x-Q24.x
Systemic lupus erythematosus	710.0x	M32.x
Human immunodeficiency virus	042.x, V08.x	B20,Z21
Multiple gestation	V27.2–V27.8, 651.x	Z37.2x-Z37.7x,O30.x

© 2023 Fink DA et al. *JAMA Network Open.*

Comorbid Conditions	ICD-9-CM	ICD-10-CM
Substance Use Disorder	292.x,304.x, 305.2x–305.9x,	F12.10, F12.11, F12.120, F12.121, F12.122, F12.129, F12.150, F12.151, F12.159, F12.180, F12.188, F12.19, F12.20, F12.21, F12.220, F12.221, F12.222, F12.229, F12.250, F12.251, F12.259, F12.280, 'F12.288, F12.29, F12.90, F12.920, F12.921, F12.922, F12.929, F12.950, F12.951, F12.959, F12.980, F12.988, F12.99, F13.10, F13.11, F13.120, F13.121, F13.129, F13.14, F13.150, F13.151, F13.159, F13.180, F13.182, F13.188, F13.19, F13.20, F13.21, F13.220, F13.221, F13.229, F13.230, F13.231, F13.232, F13.239, F13.24, F13.250, F13.251, F13.259, F13.280, F13.281, F13.28,F13.288, F13.29, F13.90, F13.920, F13.921, F13.929, F13.930, F13.931, F13.939, F13.94, F13.950, F13.951, F13.959, F13.96, F13.980, F13.982, F13.988, F13.99, F14.10, F14.11, F14.120, F14.121, F14.122, F14.129, F14.14, F14.150, F14.151, F14.159, F14.221, F14.222, F14.229, F14.23, F14.24, F14.250, F14.251, F14.259, F14.280, F14.282, F14.288, F14.29, F14.90, F14.920, F14.921, F14.922, F14.929, F14.94, F14.950, F14.951, F14.959, F14.980, F14.982, F14.988, F14.99, F15.10, F15.11, F15.120, F15.121, F15.122, F15.129, F15.14, F15.150, F15.151, F15.159, F15.180, F15.182, F15.188, F15.19, F15.20, F15.21, F15.220, F15.221, F15.222, F15.229, F15.23, F15.24, F15.250, F15.251, F15.259, F15.280, F15.282, F15.288, F15.29, F15.90, F15.920, F15.921, F15.922, F15.929, F15.93, F15.94, F15.950, F15.951, F15.959, F15.980, F15.982, F15.988, F15.99, F16.10, F16.11, F16.120, F16.121, F16.122, F16.129, F16.14, F16.150, F16.151, F16.159, F16.180, F16.183, F16.188, F16.19, F16.20, F16.21, F16.220, F16.221, F16.229, F16.24, F16.250, F16.251, F16.259, F16.280, F16.283, F16.288, F16.29, F16.90, F16.920,

© 2023 Fink DA et al. *JAMA Network Open.*

Comorbid Conditions	ICD-9-CM	ICD-10-CM
		F16.921, F16.929, F16.94, F16.950, F16.951, F16.959, F16.980, F16.983, F16.988, F16.99, F17.203, F17.208, F17.209, F17.213, F17.218, F17.219, F17.223, F17.228, F17.229, F17.293, F17.298, F17.299, F18.10, F18.11, F18.120, F18.121, F18.129, F18.14, F18.150, F18.151, F18.159, F18.17, F18.180, F18.188, F18.19, F18.20, F18.229, F18.24, F18.250, F18.29, F18.90, F18.929, F18.94, F18.951, F18.959, F18.97, F18.980, F18.988, F18.99, F19.10, F19.11, F19.120, F19.121, F19.122, F19.129, F19.14, F19.150, F19.151, F19.159, F19.16, F19.17, F19.180, F19.182, F19.188, F19.19, F19.20, F19.21, F19.220, F19.221, F19.222, F19.229, F19.230, F19.231, F19.232, F19.239, F19.24, F19.250, F19.251, F19.259, F19.27, F19.280, F19.281, F19.282, F19.288, F19.29, F19.90, F19.920, F19.921, F19.922, F19.929, F19.930, F19.931, F19.932, F19.939, F19.94, F19.950, F19.951, F19.959, F19.96, F19.97, F19.980, F19.981, F19.982, F19.988, F19.99, F13.130, F13.131, F13.132, F13.139, F19.130, F19.131, F19.132, F19.139, F12.13, F14.13, F14.93, F15.13,'F11.10','F11.13','F11.11','F11.120','F11.121','F1 1.122','F11.129','F11.14','F11.150', 'F11.151','F11.159','F11.182','F11.188','F11.19','F11.20',' F11.21','F11.220','F11.221','F11.222','F11.229','F11.23',' F11.24','F11.250', 'F11.251','F11.259','F11.281','F11.282','F11.288','F11.29' ,'F11.90','F11.920','F11.921','F11.922','F11.929','F11.93', 'F11.94','F11.950', 'F11.951','F11.959','F11.982','F11.988','F11.99'

© 2023 Fink DA et al. *JAMA Network Open.*

Comorbid Conditions	ICD-9-CM	ICD-10-CM
Alcohol abuse	291.xx, 303.xx305.0x	F10.10, F10.11, F10.120, F10.121, F10.129, F10.14, F10.150, F10.151, F10.159, F10.180, F10.181, F10.182, F10.188, F10.19, F10.20, F10.21, F10.220, F10.221, F10.229, F10.230, F10.231, F10.232, F10.239, F10.24, F10.250, F10.251, F10.259, F10.26, F10.27, F10.280, F10.281, F10.282, F10.288, F10.29, F10.920, F10.921, F10.929, F10.94, F10.950, F10.951, F10.959, F10.96, F10.97, F10.980, F10.982, F10.988, F10.99
Tobacco use	305.1.x, 649.0x	Z72.0,O99.33x,F17.x
Cardiac valvular disease	394.x–397.x, 424.x	I05-I09,I34.x
Chronic congestive heart failure	428.22, 428.23, 428.32, 428.33, 428.42, 428.43	I50.2, I50.3, I50.4
Asthma	493.x	J45.x
Preexisting diabetes mellitus	250.x, 648.0x	E10.x, E11.x
Gestational diabetes mellitus	648.8x (without pre-existing diabetes)	O24.4x,Z86.32
Obesity	278.0x,646.1x, 649.1x	O26.x,O99.21x,E66.x
Cystic fibrosis	277.0x	E84.x
Previous cesarean delivery	654.2x	O34.2x

© 2023 Fink DA et al. *JAMA Network Open.*

eTable 4. Rates of Adjusted Mortality for American Indian Patients with 95% Poisson CIs

Year	Quarter	N of Total Discharges	Adjusted Mortality/100 000 Discharges	95% Confidence Interval (Poisson)	
2008	1	781	34.8	30.1	39.5
	2	918	37.4	33.2	41.5
	3	1 003	42.2	38.2	46.2
	4	910	40.9	36.6	45.3
2009	1	1 333	27.9	25.4	30.3
	2	1 334	29.9	27.3	32.4
	3	1 418	33.7	31.1	36.2
	4	1 413	32.7	30.1	35.2
2010	1	1 339	22.4	20.2	24.6
	2	1 419	24.0	21.9	26.1
	3	1 540	27.0	24.9	29.1
	4	1 448	26.2	24.0	28.4
2011	1	1 488	18.1	16.3	19.9
	2	1 532	19.3	17.6	21.1
	3	1 756	21.7	20.1	23.3
	4	1 717	21.1	19.4	22.7
2012	1	1 779	14.7	13.3	16.0
	2	1 774	15.7	14.3	17.0
	3	1 947	17.5	16.2	18.9
	4	1 840	17.0	15.6	18.4
2013	1	2 047	11.9	10.9	12.9
	2	1 929	12.7	11.6	13.9
	3	2 128	14.2	13.1	15.3
	4	1 955	13.8	12.6	15.0
2014	1	2 068	9.7	8.8	10.6
	2	1 641	10.3	9.1	11.6
	3	1 788	11.6	10.4	12.7

© 2023 Fink DA et al. *JAMA Network Open.*

	4	1 903	11.2	10.1	12.3
2015	1	2 059	7.9	7.1	8.7
	2	1 797	8.4	7.4	9.4
	3	1 969	9.4	8.4	10.4
	4	1 828	9.2	8.1	10.2
2016	1	1 739	6.5	5.5	7.4
	2	1 666	6.9	5.9	7.9
	3	1 857	7.7	6.7	8.6
	4	1 769	7.5	6.5	8.4
2017	1	1 837	5.3	4.5	6.0
	2	1 851	5.6	4.8	6.4
	3	1 846	6.3	5.4	7.1
	4	1 711	6.1	5.2	7.0
2018	1	1 633	4.3	3.5	5.1
	2	1 676	4.6	3.8	5.4
	3	1 867	5.1	4.4	5.8
	4	1 761	5.0	4.2	5.7
2019	1	1 762	3.5	2.8	4.1
	2	1 780	3.7	3.0	4.4
	3	1 911	4.2	3.5	4.8
	4	1 625	4.0	3.3	4.8
2020	1	1 547	2.8	2.2	3.5
	2	1 585	3.0	2.3	3.7
	3	1 671	3.4	2.7	4.1
	4	1 649	3.3	2.6	4.0
2021	1	1 504	2.3	1.7	2.9
	2	1 526	2.5	1.8	3.1
	3	1 590	2.7	2.1	3.4
	4	1 378	2.7	1.9	3.4

© 2023 Fink DA et al. *JAMA Network Open.*
