# Supplementary material for: Trends in Maternal Mortality and Severe Maternal Morbidity During Delivery-Related Hospitalizations in the United States, 2008 to 2021
Source: JAMA Netw Open. 2023 Jun 22;6(6):e2317641. doi: 10.1001/jamanetworkopen.2023.17641 (PMC10288331; doi:10.1001/jamanetworkopen.2023.17641)
Supplement: Supplement 2. — Data Sharing Statement [file jamanetwopen-e2317641-s002.pdf]

Data Sharing Statement

Fink. Trends in Maternal Mortality and Severe Maternal Morbidity During Delivery-Related Hospitalizations in the United States, 2008 to 2021. *JAMA Netw Open*. Published June 22, 2023. doi:10.1001/jamanetworkopen.2023.17641

Data
Data available: No

Additional Information
Explanation for why data not available: Can not make this data available to others due to proprietary assets of Premier Inc. and HHS.
